# Completing the view – histologic insights from circular AAA specimen including 3D imaging: A methodologic approach towards histologic analysis of circumferential AAA samples

**DOI:** 10.1186/s13000-023-01359-z

**Published:** 2023-06-12

**Authors:** Anna-Leonie Menges, Maja Nackenhorst, Johannes R. Müller, Marie-Luise Engl, Renate Hegenloh, Jaroslav Pelisek, Ellen Geibelt, Anja Hofmann, Christian Reeps, Gabor Biro, Hans-Henning Eckstein, Alexander Zimmermann, Derek Magee, Martin Falk, Nadja Sachs, Albert Busch

**Affiliations:** 1 Department for Vascular Surgery, University Hospital Zurich, Zurich, Switzerland; 2 Department of Pathology, Medical University of Vienna, Vienna, Austria; 3 DFG Cluster of Excellence “Physics of Life”, TU Dresden, Dresden, Germany; 4 Technical University Munich, Department for Vascular and Endovascular Surgery, Klinikum Rechts der Isar, Munich, Germany; 5 Light Microscopy Facility, Center for Molecular and Cellular Bioengineering (CMCB), Technische Universität Dresden, Dresden, Germany; 6 Department for Visceral-, Thoracic and Vascular Surgery, Medical Faculty and University Hospital Carl Gustav Carus, TUD Dresden University of Technology, Fetscherstrasse 74, Dresden, Germany; 7 German Center for Cardiovascular Research (DZHK), Munich Heart Alliance, Berlin, Germany; 8 HeteroGenius Limited, Leeds, UK; 9 School of Computing, University of Leeds, Leeds, UK; 10 Scientific Visualization Group, Department of Science and Technology (ITN), Linköping University, Linköping, Sweden

**Keywords:** Abdominal Aortic Aneurysm, Aneurysm sac, 3D histology, Inflammation

## Abstract

Abdominal aortic aneurysm (AAA) is a pathologic enlargement of the infrarenal aorta with an associated risk of rupture. However, the responsible mechanisms are only partially understood. Based on murine and human samples, a heterogeneous distribution of characteristic pathologic features across the aneurysm circumference is expected. Yet, complete histologic workup of the aneurysm sac is scarcely reported. Here, samples from five AAAs covering the complete circumference partially as aortic rings are investigated by histologic means (HE, EvG, immunohistochemistry) and a new method embedding the complete ring. Additionally, two different methods of serial histologic section alignment are applied to create a 3D view. The typical histopathologic features of AAA, elastic fiber degradation, matrix remodeling with collagen deposition, calcification, inflammatory cell infiltration and thrombus coverage were distributed without recognizable pattern across the aneurysm sac in all five patients. Analysis of digitally scanned entire aortic rings facilitates the visualization of these observations. Immunohistochemistry is feasible in such specimen, however, tricky due to tissue disintegration. 3D image stacks were created using open-source and non-generic software correcting for non-rigid warping between consecutive sections. Secondly, 3D image viewers allowed visualization of in-depth changes of the investigated pathologic hallmarks. In conclusion, this exploratory descriptive study demonstrates a heterogeneous histomorphology around the AAA circumference. Warranting an increased sample size, these results might need to be considered in future mechanistic research, especially in reference to intraluminal thrombus coverage. 3D histology of such circular specimen could be a valuable visualization tool for further analysis.

**Supplementary Information:**

The online version contains supplementary material available at 10.1186/s13000-023-01359-z.

## Introduction

Abdominal aortic aneurysm (AAA) is the most frequent aortic aneurysm bearing an inherent threat of rupture [12]. Currently, only surgical aneurysm exclusion by either open or endovascular repair at a specific diameter threshold corresponding to an estimated annual rupture risk is an accepted treatment [8, 38]. Below this diameter, watchful waiting is indicated and past as well as current attempts for medically induced AAA growth inhibition have proven unsuccessful in clinical trials [21, 24].

This might be due to a still incomplete understanding of the underlying pathogenesis and mechanisms involved in aneurysm initiation and progression [13, 29]. While specific features, such as angiogenesis and matrix degradation in the aneurysmatic vessel wall, have been identified as key drivers of aneurysm progression in general—histologic studies have revealed a broad heterogeneity of the disease between individual patients [5, 7, 26]. Recently, it has become obvious that intraluminal thrombus (ILT) coverage providing a viscoelastic and enzymatically active compartment with eventual uneven biomechanical force distribution might be associated with a distinct histomorphologic apparel [3, 14].

However, intraoperative sampling of AAA specimens is most frequently restricted to the left-anterior and anterior wall of the aneurysm sacincised during open repair. Thus, mechanisms at the actual rupture site, as well as the other parts of the aneurysm circumference, can only be speculated on and are scarcely reported [10, 39]. Despite consensual guidelines on preparation, nomenclature and diagnostic criteria for histopathologic reports on aortic specimen, initially developed for the ascending aorta, the variety of features reported is huge and most often not standardized [16, 34, 35].

Classic histology is restricted to a 2D examination of limited numbers of 1–4 µm thicksections, only partially representing the entire specimen [26, 28]. Here, a volumetric visualization, applying 3D reconstructions of histology based whole slide image scans might be helpful for better visualization enabling a more in-depth analysis [15, 23].

Thus, in this small exploratory study we aimed to proof feasibility of histologic preparation and staining of complete AAA rings and 3D histology and provide histologic insights from the complete circumference, especially the most often neglected dorsal part of the AAA.

## Patients, material and methods

### Patient identification and ethical statement

**Fig. 1:**
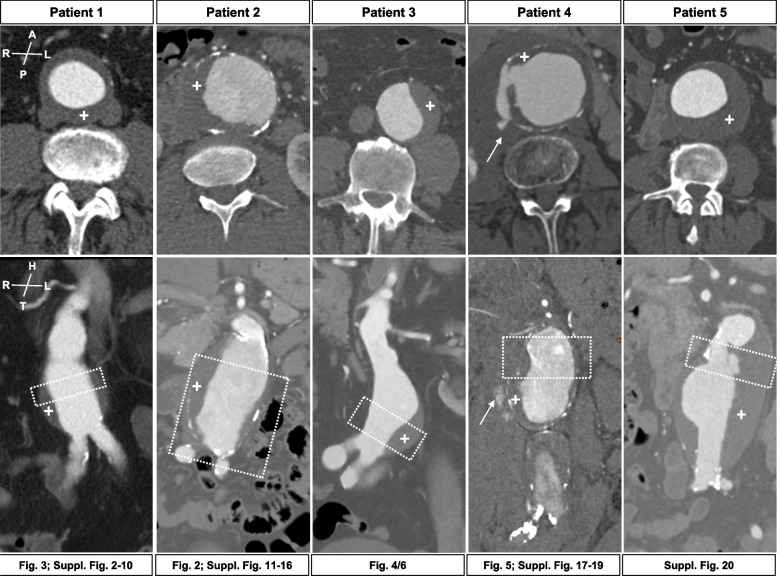
CT-angiograms and sample acquisition site. The figure presents transverse (upper panel) and coronal (lower panel) representative images of patients 1–5. The dotted box represents the approx. site of sample acquisition. Of note, ruptures site in Patient 4 (arrow). All samples are presented in the same orientation (A = anterior, P = posterior, R = right, L = left, H = head, T = tail). Of note, intraluminal thrombus (ILT) ( +) is observed to various extents in all patients. Below the CTA images, the corresponding further histologic presentation in the following (supplement) figures for each individual patient is given. Dimensions of macroscopic samples are listed in Table 1

Patient samples were acquired at two university hospitals during 2018 to 2020 from open aortic repair procedures, where anatomically and surgically feasible without posing additional threat to the respective patient. We aimed to take samples perpendicular to the aneurysm centerline axis (Fig. 1). In all cases the aneurysm sac was closed over the proximal anastomosis where possible to prevent direct contact of the duodenum and the prosthesis. Additionally, the retroperitoneum was closed to prevent direct contact of the intestine and the prosthesis. Indications for open repair were surgical reasons, patient will or operator’s choice in line with international guidelines [38].

Patient data was pseudonymized for biobanking and anonymized for further analysis. The study was performed in accordance with the declaration of Helsinki and tissue sampling was approved by the local ethics committee (*Ethikkommission Klinikum rechts der Isar*: 576/18 S and *Ethikkommission Universitätsspital Zürich*: 2020–00378).

Patient demographics and comorbidities (age, gender, arterial hypertension, smoking status, peripheral arterial disease, coronary artery disease, hyperlipidemia, diabetes, chronic obstructive pulmonary disease, dialysis or renal insufficiency) were retrieved from electronic patient records.

### Sample acquisition and preparation

After removal from the intraoperative situs, tissue was immediately placed in phosphate-buffered saline for transportation into the laboratory.

#### Classic approach (patients 1 and 2)

Samples were then fixed in formalin (4% PFA) for 24 h. If necessary, decalcification on EDTA basis (Entkalker soft SOLVAGREEN®, Carl ROTH, Karlsruhe, Germany) was performed for 2–7 days. Afterwards specimens were prepared for paraffin embedding in standard size (40 × 28 × 6.8 mm) POM histology cassettes (Kartell, Noviglio, Italy).

#### Entire aortic ring approach (patients 3–5)

The circumference of the aneurysm sac was reconstructed using a Prolene 5 0 (Ethicon) suture. Due to the expected tissue shrinkage, samples were mounted on a conical shaped hand-crafted polystyrene cylinder and pinned down with 20G needles in a fashion allowing longitudinal movement of the sample on the cylinder (Suppl. Fig. 1). The cylinder contained additional canals to enable adequate formalin penetration. After fixation and eventual decalcification (s. above), the sample was cut into approx. 6 mm thick rings with the cylinder and embedded in large (75 × 52 × 15 mm) histology cassettes (Engelbrecht, Edermünde, Germany). The suture necessary to enable adequate fixation and sectioning, was removed before paraffinization.

### Histologic staining

Sections of paraffin-embedded samples (2 µm for classic samples, 3 µm for ring samples) were mounted on glass slides (Menzel SuperFrost, 76 × 26 × 1 mm, Fisher Scientific, Schwerte, Germany for standard size; 52 × 72 × 1 mm, Engelbrecht, Edermünde, Germany for ring approach). Hematoxylin–eosin (HE) (ethanolic eosin Y solution, Mayer’s acidic hemalum solution, Waldeck, Münster, Germany) as well as Eelastica van Gieson (EvG) (picrofuchsin solution after Romeis 16th edition, Weigert’s solution I after Romeis 15th edition) stainings were accomplished according to the manufacturer’s protocol. Slides were covered using Pertex (Histolab products, Askim, Sweden) as mounting medium and glass coverslips (24 × 50 mm for standard size, 50 × 60 mm for ring approach, Engelbrecht, Edermünde, Germany).

### Immunohistochemistry

Formalin-fixed, paraffin-embedded (FFPE) sections used for immunohistochemistry were mounted on poly-l-lysine (Merck, Darmstadt, Germany) pretreated glass slides for better attachment (classic approach and ring approach) or untreated (ring approach). The sections were incubated over night at 60 °C, followed by de-paraffinization. Demasking of the antibody binding sites was achieved by cooking for 7 min in citrate acid (pH 6), made by dissolving citric acid monohydrate (Carl Roth, Karlsruhe, Germany) in distilled water. After every following step the samples were washed in Tris-buffer (Trizma base, NaCl, Merck, Darmstadt, Germany). Endogenous peroxidase activity was quenched by incubating for 15 min with 3% hydrogen peroxide (Merck, Darmstadt, Germany). Subsequently, the sections were incubated with the respective primary antibody (Suppl. Table 1). Dako REAL Antibody Diluent (Dako, Glosirup, Denmark) was used for antibody dilution. Target staining was done by incubating the samples for 25 min with the biotinylated secondary antibody, followed by incubation of 25 min adding streptavidin peroxidase and additional incubation for 2–3 min with DAB + chromogen, diluted in horseradish peroxidase substrate buffer (Dako REAL Detection System Peroxidase/DAB + , Rabbit/Mouse Kit; Dako, Glosirup, Denmark). Counterstaining was done with Mayer’s hemalum solution (Carl Roth, Karlsruhe, Germany). The sections were dehydrated and subsequently covered as described above. Especially for the large tissue sections of the aortic ring approach, detachment can occur to a certain extent, mostly during the demasking, most likely due to a greater surface. For our ring specimens, pretreatment of slides with poly-l-lysine, incubation of sections for 48 h at 60 °C, staining immediately after sectioning, and careful handling of specimens was the best and most reliable method (data not shown).

All antibodies have been used and validated in our lab before, also on aortic samples [5, 25]. Antibody specificity is routinely tested on tonsil samples and evaluated by a pathologist. Here, control incubations were performed with secondary antibody only (data not shown).

### Digital slide scanning and semi-quantitive histology

Slides (including immunohistochemistry) were then scanned with Aperio AT2 (Leica, Wetzlar, Germany), and pictures were taken with the Aperio ImageScope software (Leica). For large slides, an AxioScan.Z1 (Zeiss, Oberkochen, Germany) microscope using a Plan-Apochromat 10x/0.45 (Zeiss) objective and a HV-F202SCL (Hitatchi, Tokio, Japan) camera was available. Scanned slides were analyzed and prepared for composite figures using QuPath-0.3.2 open-source software [1]. Each cutout presented in the respective composite figure aims to cover at least one view from the luminal surface (eventual thrombus) to the adventitia. Wall thickness of fixed samples was measured with QuPath in 3–4 locations with macroscopic maximum diameter covering a perpendicular length from the beginning of the adventitia (peri-adventitial fatty tissue to collagen margin) to the supposed end of the cellular wall. The mean ± one standard deviation is shown in Table 1. Additionally, AHA classification for atherosclerosis was used to describe the amount of atherosclerosis [33].

**Table 1:** Patients’ characteristics and indication for open repair

**ID**				**Indication** for OR	**comorbidities**										**sample specifications**			
	sex	Age@OR (years)	Dmax@OR (mm)		hypertension	dyslipidemia	CAD	COPD	PAOD	dialysis	CKD	smoker (current/ex)	diabetes	aspirin intake	sample size (unfixated) (mm) (length x width x height)	wall thickness (CTA) (mm)	wall thickness (fixated) (Qupath) (mm)	AHA classification
**1**	m	70	57	iAAA	x	x						x		x	60 × 58x10	1.2	0.5 ± 0.2	6
**2**	m	59	55	iAAA	x							x			62 × 57x105	1.1	0.6 ± 0.05	7
**3**	m	74	60	iAAA		x						x	x	x	65 × 60x15	0.9	0.6 ± 0.1	5
**4**	f	79	72	rTAAA (type IV)	x			x				x		x	80 × 75x20	1.2	0.8 ± 0.2	7
**5**	m	79	75	iAAA	x	x				x		x		x	76 × 76x17	1.0	0.7 ± 0.1	6

*m* male, *f* female, *OR* Open repair; intact (i) or ruptured (r) abdominal aortic aneurysm (AAA), *TAAA* Thoraco-abdominal aortic aneurysm type IV Crawford classification, *CAD* Coronary artery disease, *COPD* Chronic obstructive pulmonary disease, *PAOD* Peripheral arterial occlusive disease, *CKD* Chronic kidney disease with creatinine > 1.2 mg/dl, *CTA* Computed tomography angiography; QuPath wall thickness mean ± SD; *AHA* American Heart Association

### Section alignment and 3D histology image acquisition

Serial sections from Patients 3 and 4 were scanned and aligned digitally using the HeteroGenius MIM Multi-stain Add-on (HeteroGenius, Leeds, UK) to form two 3D image stacks [32]. This add-on initially estimates relative rotation and translation between sequential sections and corresponding sections of different stains, before correcting for non-rigid warping caused by the sectioning process. The HE stained stack was used as the reference stack, and the EvG stack was used as the secondary stack, with the software aligning all images in the reference stack first, before aligning the secondary images to their corresponding primary image. The aligned sections were exported at full resolution in SVS format for visualization in the open-source software Inviwo [15, 18]. The white background of the slide images was turned transparent to reveal the three-dimensional structure of the slide stack. Additional depth cues are provided by adding a dark tint to the transparent regions.

Additionally, as second method, we attempted the 3D reconstruction of histological samples using exclusively open source software. Therefore, a subset of histological sections were first aligned with an arbitrarily chosen central section of the available stack using a registration workflow [9]. Briefly, this applied Big Warp registration toolbox and Fiji (v 1.53q) for the alignment as well as QuPath (v4.0) for the data handling and image transformations. The data was then exported as separate images in tif-format and concatenated along the z-axis. We finally used Napari to generate a freely navigable rendering of the histological data stack [31].

## Results

Five circumferential AAA patient samples were available for further analysis, thereof one ruptured and four elective cases. Circumferential ILT was present in every AAA (Fig. 1). Patient details and indications for open repair are shown in Table 1. None of the patients had connective tissue disease or showed any such stigmata. All patients were successfully operated and could be discharged from hospital after 16.6 ± 10.6 days. No aneurysm related bleeding complication occurred (data not shown).

**Fig. 2:**
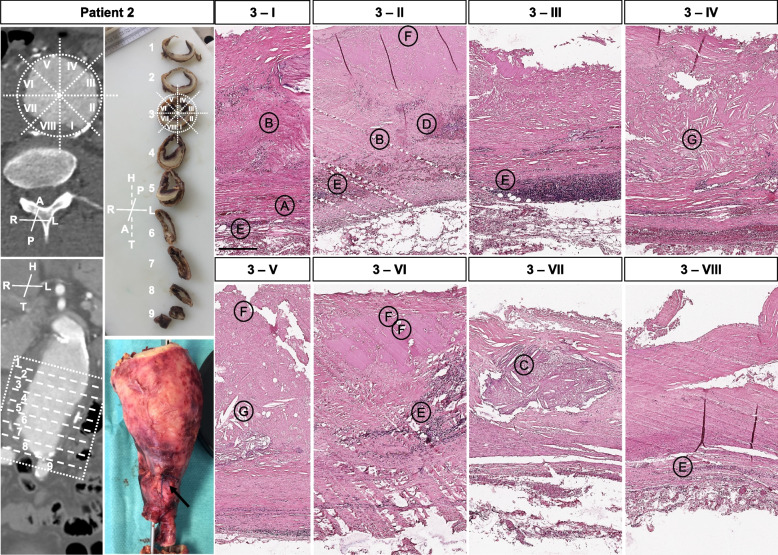
Patient 2 sample acquisition, cutting scheme and histomorphology. CT-angiogram and photo of the un-fixed specimen and sequentially cut formalin fixed sample demonstrate the approx. acquisition site. The dotted box and circle depict the specific termination for samples (1–9; I-VIII). Histologic photos show eight (I-VIII) representative whole wall cutouts at level 3 (HE staining). Specific histologic features observed include: **A** fragmented elastic fibres, **B** collagen deposition/medial fibrosis, **C** calcification, **D** intramural bleeding, **E** mononuclear inflammatory cell infiltration **F** thrombus coverage, and **G** cholesterol clefts. Overall, disorganization of vascular smooth muscle cells and lamellar units is seen to various extents. All samples are presented in the same orientation (A = anterior, P = posterior, R = right, L = left, H = head, T = tail). For all histologic photos the aortic lumen is oriented upwards (scale bar 250 µm)

Sample acquisition covered rings perpendicular to the aneurysm centerline from the proximal or distal part of the aneurysm sac (Fig. 1, Suppl. Fig. 1). For patient 2, the complete middle and distal section of the aneurysm including the aortic bifurcation was available (Fig. 2). For standard histology and staining, no major problems were observed during the slide production. However, immunohistochemistry was technically challenging for aortic rings, especially during epitope retrieval due to tissue/slide disintegration (Suppl. Figs. 18–20).

**Fig. 3:**
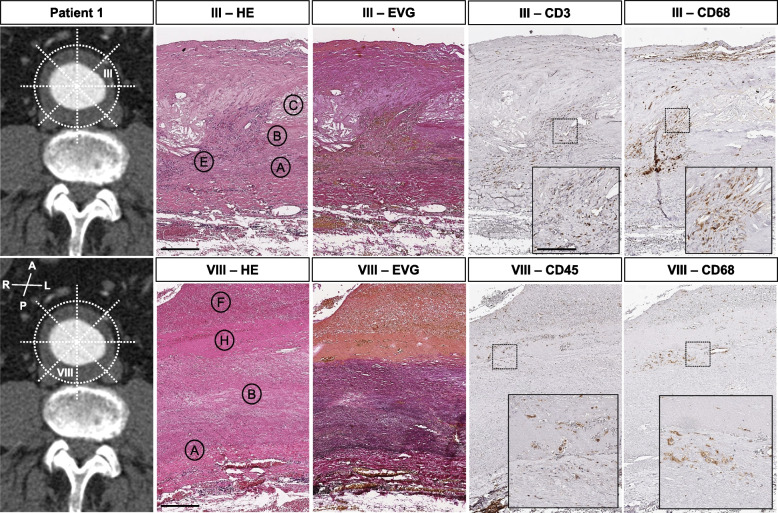
Patient 1 sample acquisition, histomorphology and immunohistochemistry left anterior wall (III) and dorsal wall (VIII). CT-angiogram and dotted lines demonstrate the approx. site of samples acquisition. Histologic and immunohistochemistry photos show a representative cutout from the same position of the specimen for HE, EVG and various antibody stainings. Specific histologic features observed include: **A** fragmented elastic fibers, **B** collagen deposition/medial fibrosis, **C** calcification, **E** mononuclear inflammatory cell infiltration, **F** thrombus coverage and **H** mucoid deposition. CD 68 positive cells are seen around the intramural plaque (upper panel) but also in the ILT (lower panel). CD 3 positive cells are located in proximity to the plaque, whereas CD45 positive cells are seen in and close to the ILT. For all histologic photos the aortic lumen is oriented upwards. All samples are presented in the same orientation (A = anterior, P = posterior, R = right, L = left, H = head, T = tail) (scale bar 200 µm; cutouts 50 µm)

**Fig. 4:**
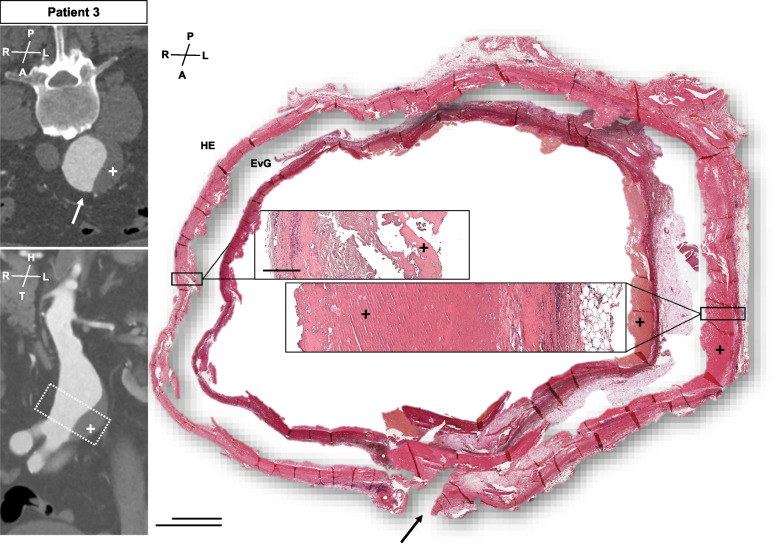
Patient 3 sample acquisition and histomorphology. CT-angiogram and dotted lines demonstrate the approx. site of samples acquisition. The arrow marks the aneurysm sac incision during open repair. The histologic photos show a complete circular preparation of the aneurysm sac for HE (outer picture) and EVG (inner picture). Samples are oriented in line with the CT-angiogram. The left side of the aneurysm is covered with notable ILT **( +)**, whereas only minimal ILT seen on the right part of the aneurysm sac. The histomorphology comparing both sides of the aneurysm sac appears similar (cutouts) (A = anterior, P = posterior, R = right, L = left, H = head, T = tail) (scale bar 5 mm each, upper bar = inner photo)

**Fig. 5:**
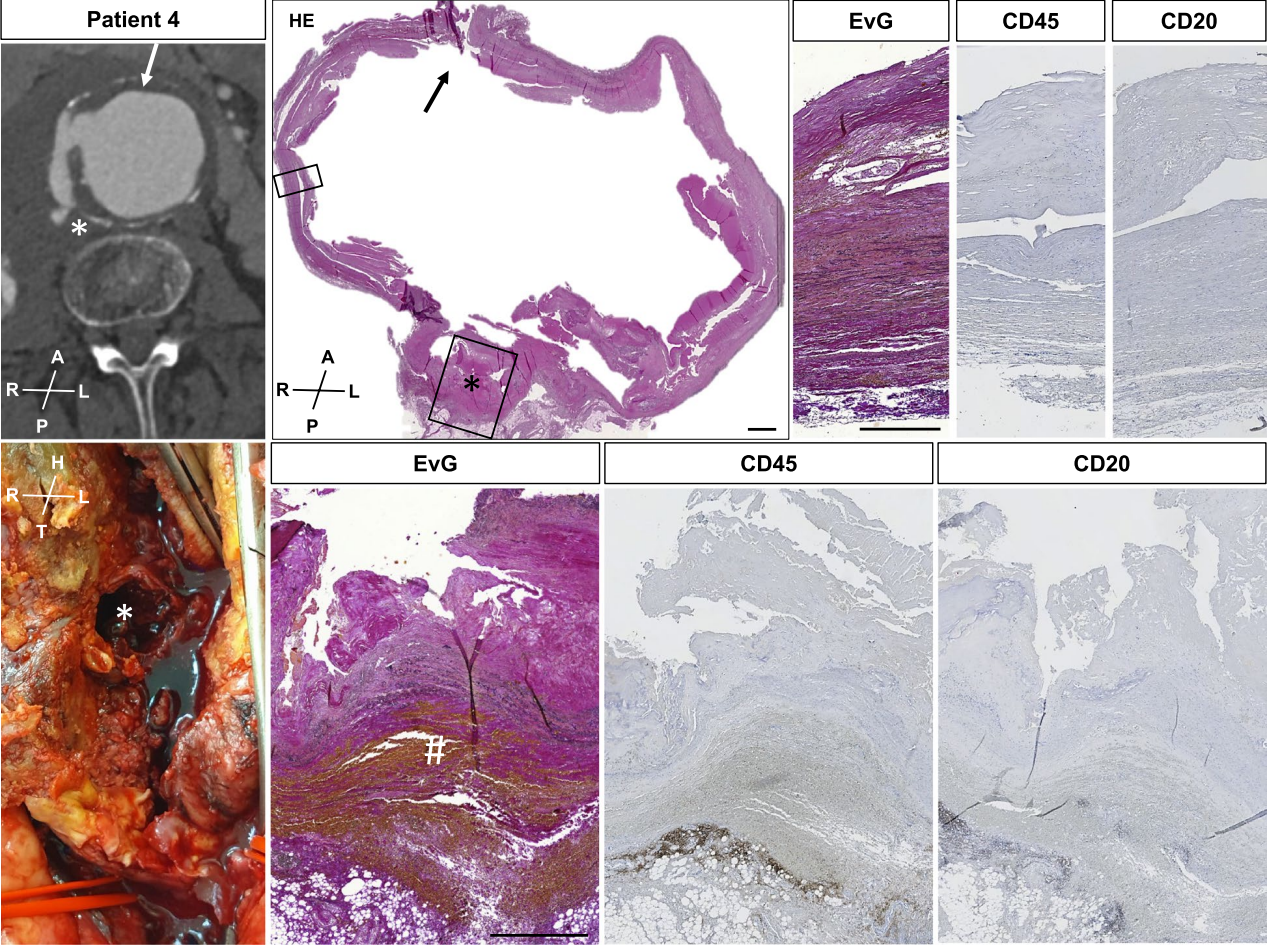
Patient 4 sample acquisition, rupture site and histomorphology. CT-angiogram demonstrates the approx. site of sample acquisition. The arrow marks the aneurysm sac incision during open repair. Photo shows the intraoperative situs with the rupture site (asterisk) at the dorsal right part of the aneurysm sac (iliac arteries marked with red vessel loops. The histologic photos show a complete circular preparation of the aneurysm sac (HE) oriented in line with the CT-angiogram. Cutouts are presented from the right lateral wall (upper panel) and the rupture site (lower panel). EvG stainig demonstrates fragmented elastic fibers and matrix remodeling as described above for the lateral wall and an amorphous bleb-like structure with massive intramural hemorrhage **(#)** at the rupture site. At the outer margins of this structure, CD45 and CD20 positive cells are found in the adventitia. For all histologic photos the aortic lumen is oriented upwards. All samples are presented in the same orientation (A = anterior, P = posterior, R = right, L = left, H = head, T = tail) (scale bar 5 mm for HE; cutouts upper panel 250 µm; cutouts lower panel 500 µm)

**Fig. 6:**
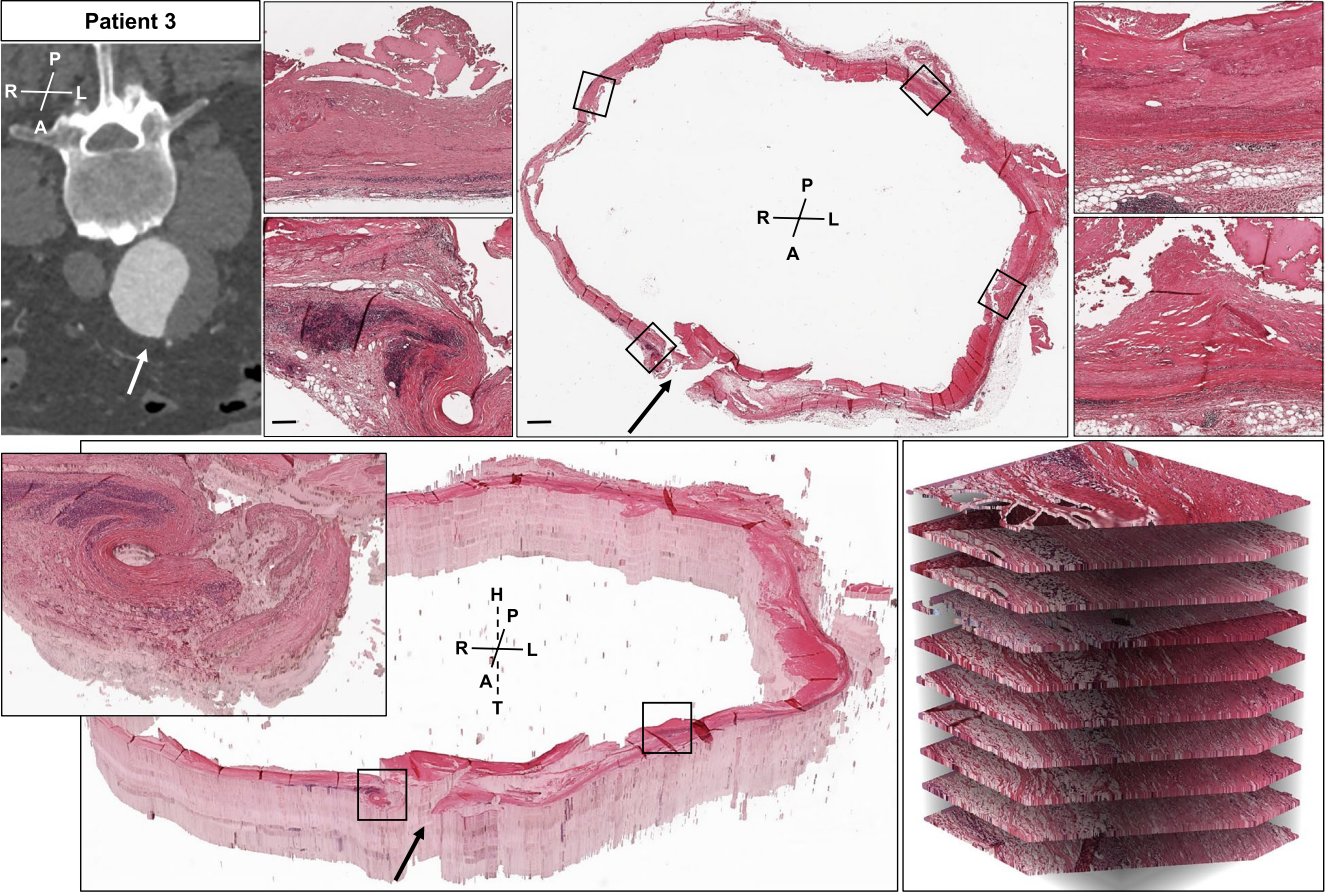
Patient 3 sample acquisition, histomorphology and 3D histology. CT-angiogram demonstrates the approx. site of samples acquisition. The arrow marks the aneurysm sac incision during open repair. The histologic photos (upper panel) show a complete circular preparation of the aneurysm sac for HE and four representative cutouts demonstrating a differing histomorphology (lumen orientated upwards). The lower panel demonstrates 3D histology HE representative images. The slide image background was removed based on color similarity to white and then set to be transparent. This enables the interactive exploration of the internal structures. Zooming in the 3D histology visualization highlights the large differences between the horizontal resolution of up to 0.25 um/pixel and the vertical axis. Samples are oriented mirrored with the CT-angiogram. (A = anterior, P = posterior, R = right, L = left, H = head, T = tail) (scale bar 2 mm for overview and 250 µm for blow-ups)

Circumferential histologic evaluation included elastic fiber loss and fragmentation. Specifically, less than 25% of the expected 20–25 layers were observed in all patients and all samples. Collagen deposition or medial fibrosis, calcification, intramural micro bleedings, inflammatory cell infiltration and eventual thrombus coverage revealed a very distinct histomorphology along the perimeter of the aneurysm sac (Figs. 2, 3, 4, 5 and 6, Suppl. Figs. 2–20). Disorganization of vascular smooth muscle cells and lamellar units was seen to a great extent, with no obvious distribution pattern of morphologic features for i.e. anterior vs. posterior aneurysm wall in all patients investigated. This was similarly observed for patient 2 in longitudinal orientation (Fig. 2, Suppl. Figs. 11–16). A clearly detectable intima was absent in most samples, where ILT was visible to various extents. Mucoid depositions were seen in some samples adjacent to the thrombus (Figs. 2 and 4). Similar aneurysm sac morphology was seen at various ILT coverage (Figs. 1, 4 and 6). Inflammatory and immune-cell visualization equally showed a heterogeneous distribution of i.e. CD3, CD68 and CD45 positive cells along the circumference (Figs. 3 and 5, Suppl. Figs. 3–10, 18, 19). However, histology at the potential rupture site in patient 4 was distinct from the remaining perimeter in this unique sample showing intramural hemorrhage and CD45/CD20 positive adventitial infiltrates (Fig. 5, Suppl. Figs. 17–19).

In our subjective perception, analysis and visualization were easier using complete circular samples made digitally available (Figs. 4, 5 and 6, Suppl. Figs. 17–20). For patients 1 and 4, immunohistochemistry for various inflammation-related epitopes emphasized these impressions (Figs. 3 and 5, Suppl. Figs. 3–10, 18, 19, Suppl. Table 1).

Finally, the consecutive aortic ring alignements are shown for 3D histology. This provided an illustrative amendment to the observations described above, emphasizing the distinct circumferential histomorphology in all samples investigated (Fig. 6).

## Discussion

To our best knowledge, this study shows for the first time a detailed histomorphology of circular AAA specimen and explored the possibility of embedding complete aortic rings from intraoperative human samples. Here, we demonstrate a very heterogeneous appearance around the aneurysmatic circumference (Figs. 2, 3, 4, 5 and 6, Suppl. Figs. 2–20).

This uneven histomorphologic apparel observed among individual patients is in line with previous results from the ascending aorta as well as the infrarenal aneurysmatic aorta [4, 5, 11, 17, 26]. Here, even a consensus document on diagnostic criteria exists [34, 35]. However, AAA patients are typically older than patients suffering from ascending or thoracic aortic disease and have rarely connective tissue disorders. Additionally, the infrarenal aorta has a different composition and phylogenetic background [27]. Thus histologic hallmarks might differ [29].

Additionally, a high variance of gene expression patterns has been demonstrated between i.e. tunica media and adventitia emphasizing the distinct pathologic features associated with a distinct histomorphology [6, 22]. Our images demonstrate an uneven distribution of defined histopathologic features over the entire aneurysm circumference, differently affecting the adventitial and medial layer (Figs. 2 and 5, Suppl. Figs. 18 and 25). While this intra-individual disease specificity is often considered responsible for negative results from previous and current clinical trials on AAA growth abrogation [21, 24] – one could also hypothesize that the inflicted pathomechanisms may be found in every patient at differing circumferential and longitudinal positions, yet might not always play the predominant role in the imbalance of pro-aneurysmatic pathways and potential healing responses [13, 29].

In addition, an uneven geometry of the aneurysm centerline might influence an uneven distribution of ILT affecting the histologic apparel and vice versa [3, 30]. The five patients investigated here did not show excessive bulging to either side of their respective aneurysm (Fig. 1). Also, the maximum diameters varied from 55-75 mm (Table 1). However, no correlations between histologic features and AAA diameter have been reported so far. The possible association of ILT on histologic appearance should be investigated in a larger patient cohort. In this context, experimental radiology using new radioactive tracers or specific magnetic resonance imaging probes have shown an unequal distribution of their respective target in human aortic aneurysm, both in circumferent and longitudinal direction [19, 20].

Previous reports on specific pathologic features from AAA rupture sites have demonstrated an increased local expression of matrix-metalloproteinases, however, are inconclusive about the cellular microenvironment and the potentially involved cytokines [39, 40]. The sample of patient 4 included in this study connects the potential macroscopic rupture site to a bleb in the aortic ring, which did show intramural hemorrhage and inflammatory cells positive for CD20 and CD45 (Fig. 5, Suppl. Figs. 18 and 19). However, such observations and reported results need to be considered with caution, since a standardized approach towards histologic analysis is missing for AAAs [4, 16, 26, 35]. Humoral immune cells such as B-/T-cells have been shown to reside in the aneurysm wall before (Figs. 3 and 5, Suppl. Figs. 3–10) [13, 29]. Additionally, the exact localization of sample acquisition is not defined in most studies and certainly non-standardized regarding the potential rupture site [39, 40].

These limitations also apply to our study with only five individual tissue samples included in total and one ruptured case specifically. Thus, the results reported are purely descriptive and no universal conclusions can be drawn. Most importantly, longitudinal data from the middle of the AAA to the aortic bifurcation was only available for patient 2 (Fig. 2, Suppl. Figs. 11–16). However, such information is crucially warranted since many expressions analysis focus on the differences between i.e. non-dilated aneurysm neck areas and the most dilated parts within the same patient [2, 40].

While three dimensional radiologic imaging is frequently used to study the biomechanical behavior of aortic aneurysms, 3D histology has been mostly reported to improve diagnostic accuracy and visualization in cancer specimen [37]. Both, HE and immunofluorescence for vessel imaging have been applied, [15, 36]. Technical challenges include correct tissue preparation and clearing, followed by digital slide registration and computation of 3D models with contrast between the horizontal resolution of the 2D slide images, which is 0.25 um/pixel at 40 × magnification, and the total number of images in the slide stack. Eventual correction for tissue shrinkage, misalignment and missing data between two consecutive slides and artifacts of slide preparation, i.e. different stain colors, overlapping tissue have an effect on registration and 3D histology [28].

While technically feasible, the diagnostic benefit is yet to be elucidated. However, regarding the examples provided in this study, 3D histology is of great value to improve visualization of complex intraoperative specimen by enabling the interactive exploration of the co-registered slide stack, thereby revealing three-dimensional structures also along the z-axis. We demonstrated the generation of a 3D rendering of the aligned histological sections using exclusively open-source software [31]. To improve the 3D histology, many more slides of the same sample with a better registration between consecutive slides are required to compensate for the mentioned shortcomings. Limitations are given by the dimensions of the data rather than the software used as well as the tissue disintegration during the mounting process (data not shown).

## Conclusion

This exploratory study including five circumferential detailed analyzes of human AAA specimen demonstrates heterogeneous histomorphology along the aneurysm sac perimeter. Warranting a higher number of specimen investigated, these results need to be considered in future mechanistic research and advanced imaging. 3D histology of such circular specimen is time consuming, yet feasible and could be a valuable tool for improved visualization and further analysis.

### Supplementary Information

**Additional file 1: Suppl. Table 1.** Antibody list. **Suppl. Figure 1.** Sample preparation Patient 1-5. **Suppl. Figure 2.** Patient 1 sample acquisition and HE histomorphology. **Suppl. Figure 3.** Patient 1 sample acquisition, histomorphology and immunohistochemistry I. **Suppl. Figure 4.** Patient 1 sample acquisition, histomorphology and immunohistochemistry II. **Suppl. Figure 5.** Patient 1 sample acquisition, histomorphology and immunohistochemistry III. **Suppl. Figure 6.** Patient 1 sample acquisition, histomorphology and immunohistochemistry IV. **Suppl. Figure 7.** Patient 1 sample acquisition, histomorphology and immunohistochemistry V. **Suppl. Figure 8.** Patient 1 sample acquisition, histomorphology and immunohistochemistry VI. **Suppl. Figure 9.** Patient 1 sample acquisition, histomorphology and immunohistochemistry VII. **Suppl. Figure 10.** Patient 1 sample acquisition, histomorphology and immunohistochemistry VIII. **Suppl. Figure 11.** Patient 2 sample acquisition and histomorphology 1. **Suppl. Figure 12. **Patient 2 sample acquisition and histomorphology 2. **Suppl. Figure 13.** Patient 2 sample acquisition and histomorphology 4. **Suppl. Figure 14.** Patient 2 sample acquisition and histomorphology 5. **Suppl. Figure 15.** Patient 2 sample acquisition and histomorphology 6. **Suppl. Figure 16.** Patient 2 sample acquisition and histomorphology 7. **Suppl. Figure 17.** Patient 4 sample acquisition and histomorphology. **Suppl. Figure 18.** Patient 4 sample acquisition, histomorphology and immunohistochemistry 1. **Suppl. Figure 19.** Patient 4 sample acquisition, histomorphology and immunohistochemistry 2. **Suppl. Figure 20.** Patient 5 sample acquisition and histomorphology.
