# Correction: Completing the view – histologic insights from circular AAA specimen including 3D imaging: A methodologic approach towards histologic analysis of circumferential AAA samples

**DOI:** 10.1186/s13000-023-01420-x

**Published:** 2023-12-02

**Authors:** Anna-Leonie Menges, Maja Nackenhorst, Johannes R. Müller, Marie-Luise Engl, Renate Hegenloh, Jaroslav Pelisek, Ellen Geibelt, Anja Hofmann, Christian Reeps, Gabor Biro, Hans-Henning Eckstein, Alexander Zimmermann, Derek Magee, Martin Falk, Nadja Sachs, Albert Busch

**Affiliations:** 1 Department for Vascular Surgery, University Hospital Zurich, Zurich, Switzerland; 2 Department of Pathology, Medical University of Vienna, Vienna, Austria; 3 DFG Cluster of Excellence ?Physics of Life?, TU Dresden, Dresden, Germany; 4 Department for Vascular and Endovascular Surgery, Klinikum Rechts der Isar, Technical University Munich, Munich, Germany; 5 Light Microscopy Facility, Center for Molecular and Cellular Bioengineering (CMCB), Technische Universit?t Dresden, Dresden, Germany; 6 Department for Visceral-, Thoracic and Vascular Surgery, Medical Faculty and University Hospital Carl Gustav Carus, TUD Dresden University of Technology, Fetscherstrasse 74, Dresden, Germany; 7 German Center for Cardiovascular Research (DZHK), Munich Heart Alliance, Berlin, Germany; 8 HeteroGenius Limited, Leeds, UK; 9 School of Computing, University of Leeds, Leeds, UK; 10 Scientific Visualization Group, Department of Science and Technology (ITN), Linköping University, Linköping, Sweden

**Correction: Diagnostic Pathology 18, 73 (2023)**

10.1186/s13000-023-01359-z

Following the publication of the original article [1], the authors requested to update affiliations 4 and 6 as follows:

4. Technical University Munich, Department for Vascular and Endovascular Surgery, Klinikum Rechts der Isar, Munich Germany
6. Department for Visceral-, Thoracic and Vascular Surgery, Medical Faculty and University Hospital Carl Gustav Carus, TUD Dresden University of Technology, Fetscherstrasse 74, Dresden, Germany

The original article [1] has been updated.
