# Supplementary material for: Completing the view – histologic insights from circular AAA specimen including 3D imaging: A methodologic approach towards histologic analysis of circumferential AAA samples
Source: Diagn Pathol. 2023 Jun 12;18:73. doi: 10.1186/s13000-023-01359-z (PMC10259026; doi:10.1186/s13000-023-01359-z)
Supplement: Supplementary file 1 — Additional file 1: Suppl. Table 1. Antibody list. Suppl. Figure 1. Sample preparation Patient 1-5. Suppl. Figure 2. Patient 1 sample acquisition and HE histomorphology. Suppl. Figure 3. Patient 1 sample acquisition, histomorphology and immunohistochemistry I. Suppl. Figure 4. Patient 1 sample acquisition, histomorphology and immunohistochemistry II. Suppl. Figure 5. Patient 1 sample acquisition, histomorphology and immunohistochemistry III. Suppl. Figure 6. Patient 1 sample acquisition, histomorphology and immunohistochemistry IV. Suppl. Figure 7. Patient 1 sample acquisition, histomorphology and immunohistochemistry V. Suppl. Figure 8. Patient 1 sample acquisition, histomorphology and immunohistochemistry VI. Suppl. Figure 9. Patient 1 sample acquisition, histomorphology and immunohistochemistry VII. Suppl. Figure 10. Patient 1 sample acquisition, histomorphology and immunohistochemistry VIII. Suppl. Figure 11. Patient 2 sample acquisition and histomorphology 1. Suppl. Figure 12. Patient 2 sample acquisition and histomorphology 2. Suppl. Figure 13. Patient 2 sample acquisition and histomorphology 4. Suppl. Figure 14. Patient 2 sample acquisition and histomorphology 5. Suppl. Figure 15. Patient 2 sample acquisition and histomorphology 6. Suppl. Figure 16. Patient 2 sample acquisition and histomorphology 7. Suppl. Figure 17. Patient 4 sample acquisition and histomorphology. Suppl. Figure 18. Patient 4 sample acquisition, histomorphology and immunohistochemistry 1. Suppl. Figure 19. Patient 4 sample acquisition, histomorphology and immunohistochemistry 2. Suppl. Figure 20. Patient 5 sample acquisition and histomorphology. [file 13000_2023_1359_MOESM1_ESM.zip › Supplement Material.docx]

**Supplement Material**

**Supplement Table**

| **Antibody** | **Clone** | **Manufacturer** | **Dilution** | **Target** |
| --- | --- | --- | --- | --- |
| KI67 | MIB-1 | IL Immunologic | 1:500 | cell proliferation |
| CD34 | QBEND/10 | Cell marque | 1:1000 | haematopoietic and vascular - associated progenitor cells |
| α Smooth Muscle Actin | HHF 35 | Dako | 1:200 | myofibroblast formation |
| CD3 | MRQ-39 | Cell marque | 1:500 | T-cells |
| CD20cy | L26 | Dako | 1:500 | B-cells |
| CD68 | KP1 | Dako | 1:2000 | monocytes, macrophages |
| CD45 | 2B11+PD7/26 | Dako | 1:200 | leukocytes |

**Suppl. Table 1. Antibody list.**

**Supplement Figures**

**
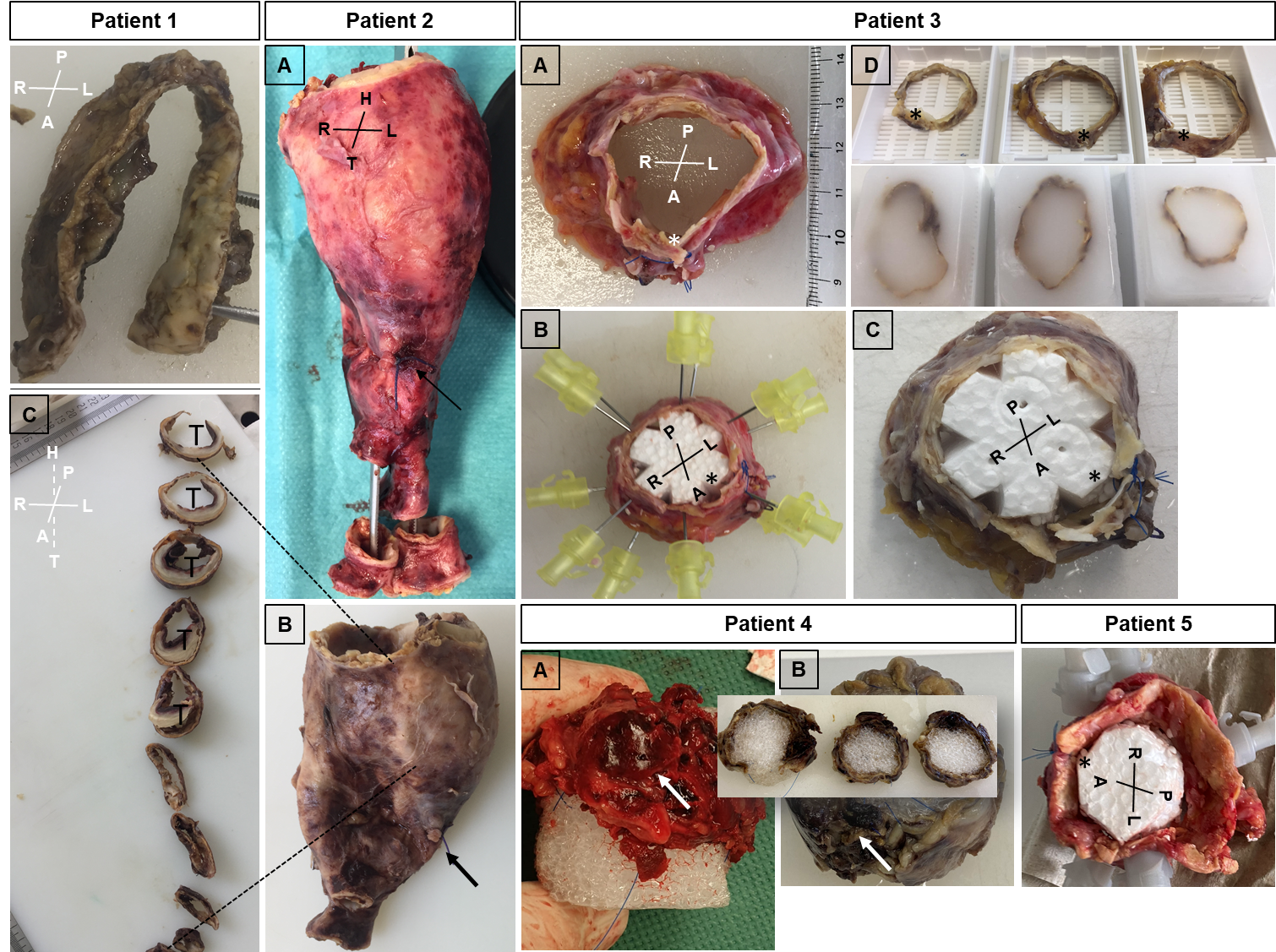
**

**Suppl. Figure 1: Sample preparation Patient 1-5.** **(Patient 1)** Sample was fixed without re-attachment of the aortic circumference after ventro-lateral incision during open repair. Sample was then divided into eight equal probes for histologic analysis. **(Patient 2)** Complete middle and distal part of the aneurysm before **(A)** and after **(B)** fixation in formaldehyde (arrow indicated the ligated inferior mesenteric artery). **(C)** Equal rings of 6 mm thickness demonstrating the unevenly distributed thrombus **(T)** coverage over the aneurysm sac. Each ring was then divided into eight equal probes for histologic analysis. **(Patient 3) (A)** The circumference of the sample was recovered by a suture **(*)** before fixation. The sample was then mounted on a handcrafted conical polystyrene cylinder and fixed with pins **(B)** allowing shrinkage along the cylinder meanwhile fixation **(C)**. The sample was then cut into 6 mm thick rings and embedded completely in paraffin using 75x52x15mm histo cassettes **(D)**. **(Patient 4)** **(A)** The circumference was reconstructed by suture **(*)** including the rupture side and fixed on a polytstyrene cylinder **(B)**. **(inlay)** For cutting equal 6mm thick rings, the polystyrene was left with the tissue and removed before paraffinization. **(Patient 5)** Sample preparation in accordance to patients 3 and 4. All samples are presented in similar orientation (A = anterior, P = posterior, R = right, L= left, H = head, T = tail).

**
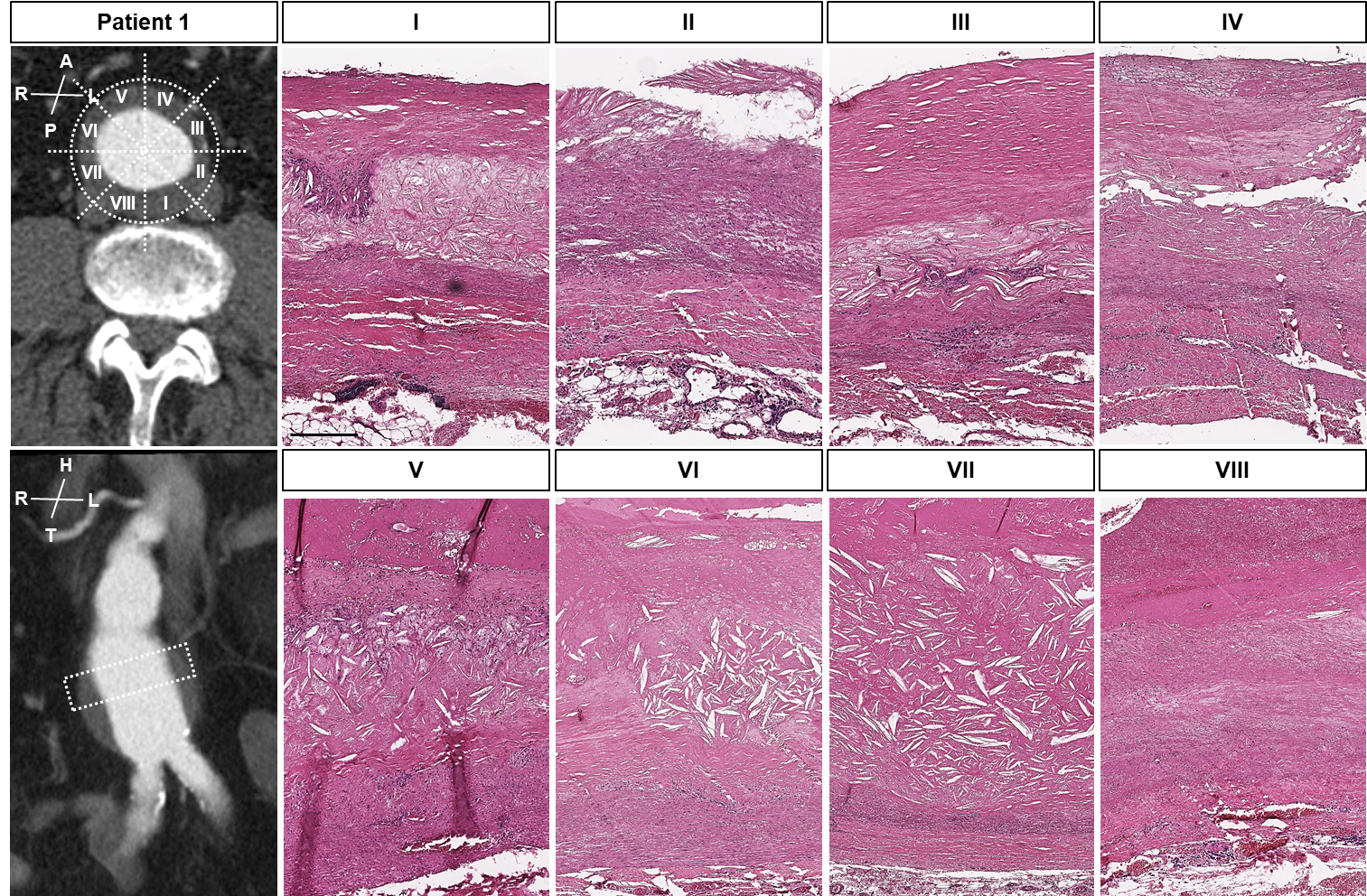
**

**Suppl. Figure 2: Patient 1 sample acquisition and HE histomorphology.** CT-angiogram and dotted lines demonstrate the approx. site of samples acquisition. Histologic images show a representative cutout from all eight aneurysms sac localizations. For all histologic images the aortic lumen is oriented upwards. All samples are presented in the same orientation (A = anterior, P = posterior, R = right, L= left, H = head, T = tail) (scale bar 200µm).

**
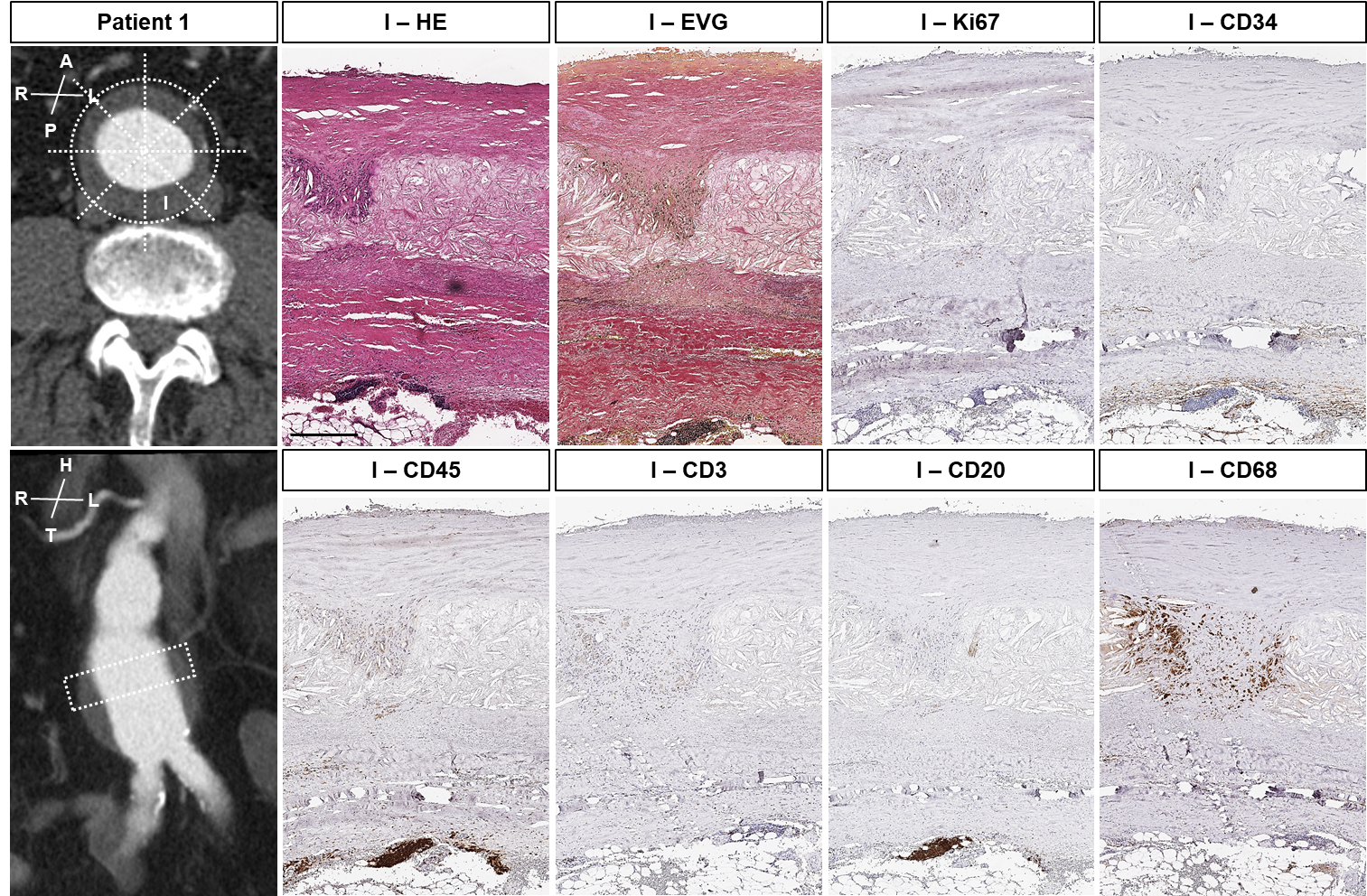
**

**Suppl. Figure 3: Patient 1 sample acquisition, histomorphology and immunohistochemistry I.** CT-angiogram and dotted lines demonstrate the approx. site of samples acquisition. Histologic and immunohistochemical scans show a representative cutout from the same position of the specimen for HE, EVG and various antibody stainings. For all histologic images the aortic lumen is oriented upwards. All samples are presented in the same orientation (A = anterior, P = posterior, R = right, L= left, H = head, T = tail) (scale bar 200µm). The antibody list can be found in **Suppl. Table I**.

**
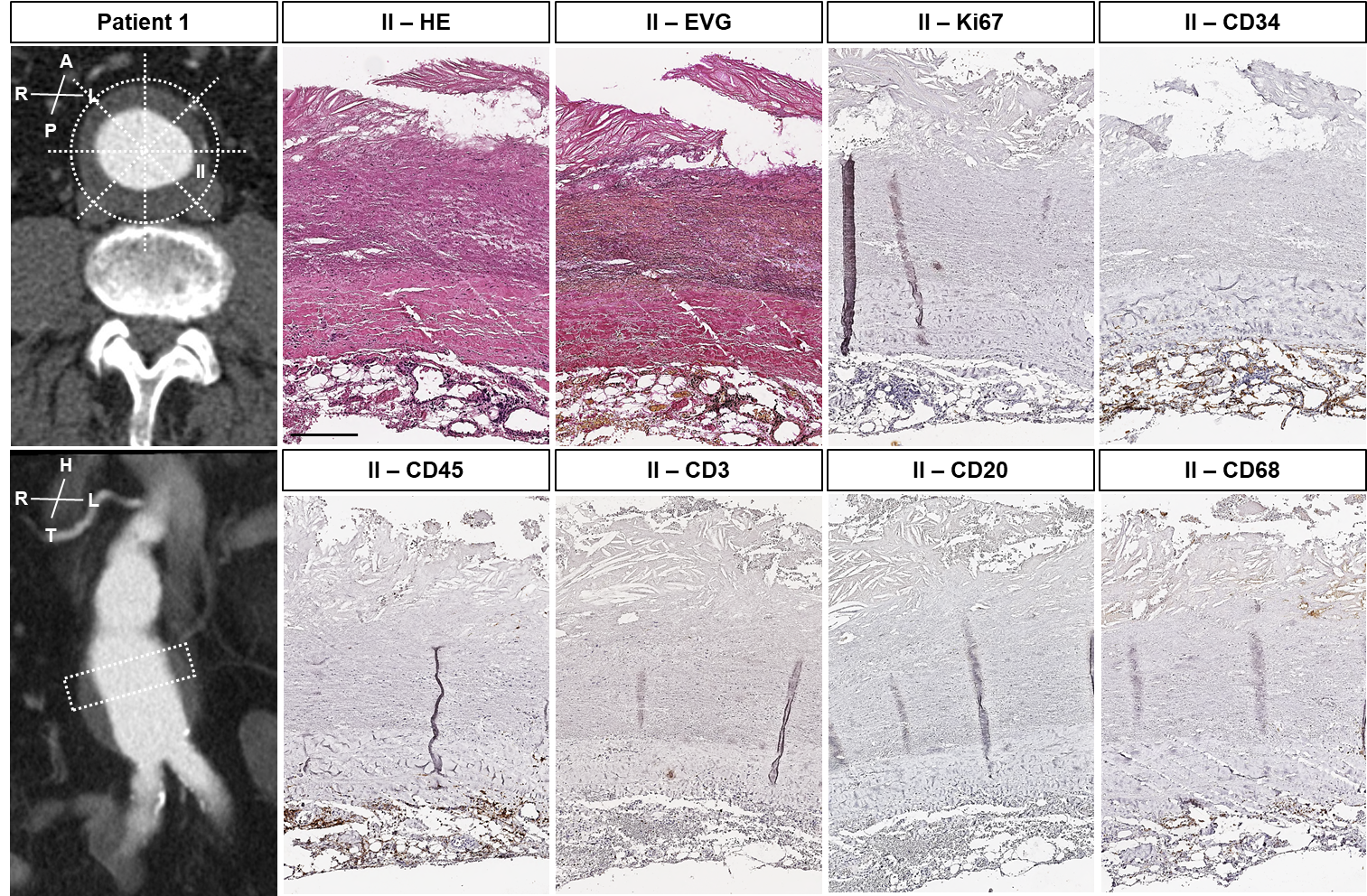
**

**Suppl. Figure 4: Patient 1 sample acquisition, histomorphology and immunohistochemistry II.** CT-angiogram and dotted lines demonstrate the approx. site of samples acquisition. Histologic and immunohistochemical scans show a representative cutout from the same position of the specimen for HE, EVG and various antibody stainings. For all histologic images the aortic lumen is oriented upwards. All samples are presented in the same orientation (A = anterior, P = posterior, R = right, L= left, H = head, T = tail) (scale bar 200µm). The antibody list can be found in **Suppl. Table I**.

**
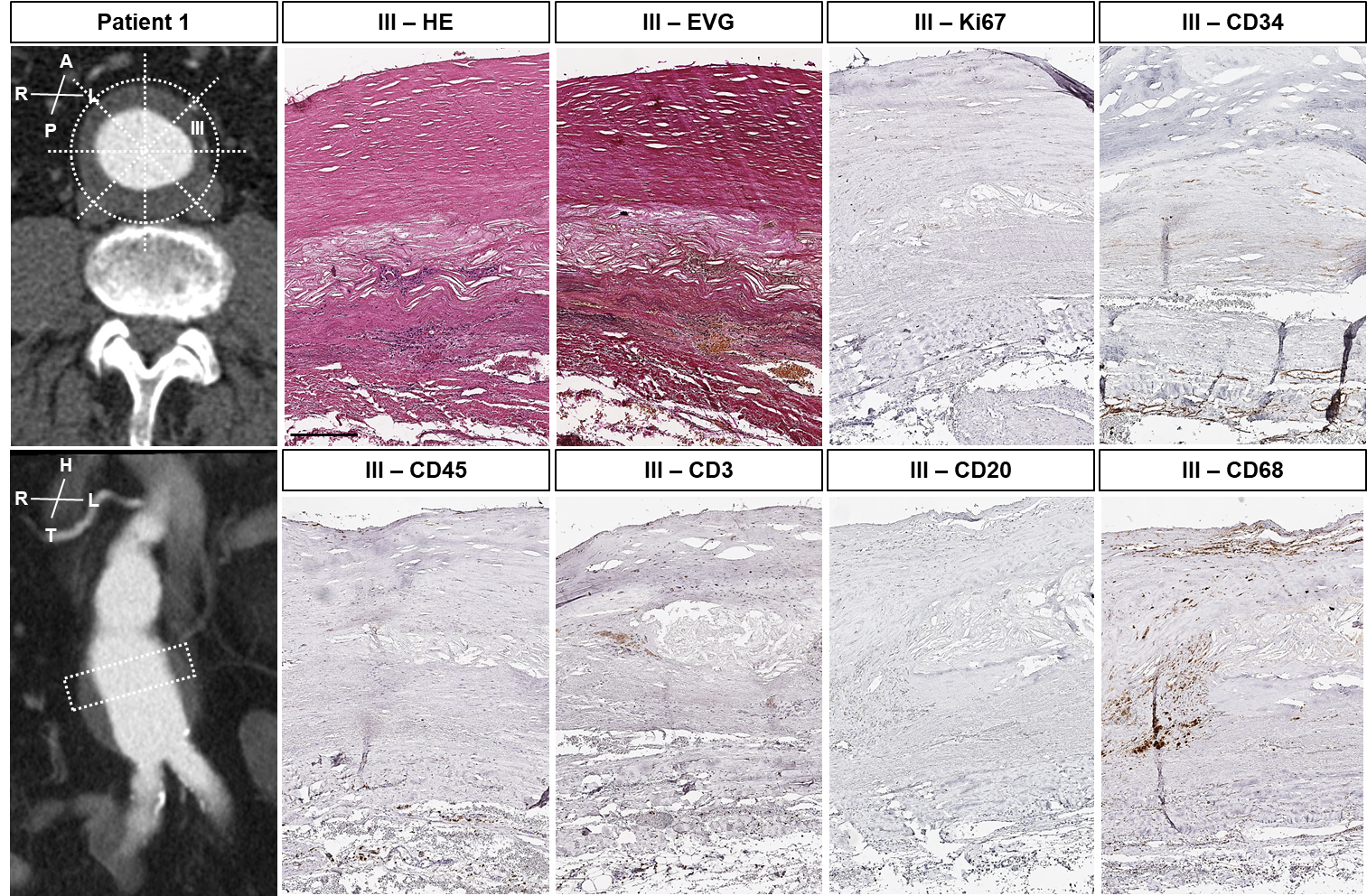
**

**Suppl. Figure 5: Patient 1 sample acquisition, histomorphology and immunohistochemistry III.** CT-angiogram and dotted lines demonstrate the approx. site of samples acquisition. Histologic and immunohistochemical scans show a representative cutout from the same position of the specimen for HE, EVG and various antibody stainings. For all histologic images the aortic lumen is oriented upwards. All samples are presented in the same orientation (A = anterior, P = posterior, R = right, L= left, H = head, T = tail) (scale bar 200µm). The antibody list can be found in **Suppl. Table I**.

**
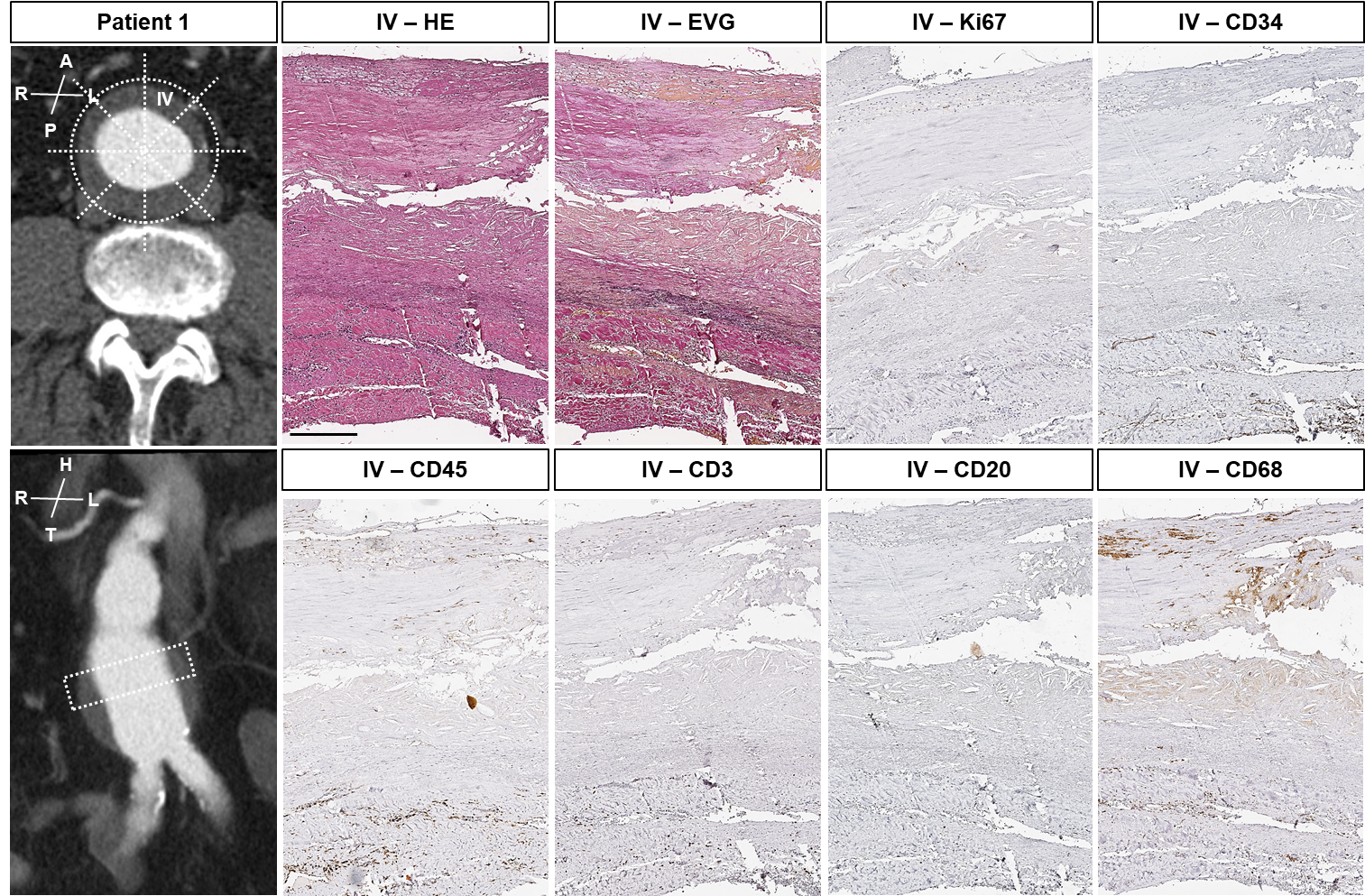
**

**Suppl. Figure 6: Patient 1 sample acquisition, histomorphology and immunohistochemistry IV.** CT-angiogram and dotted lines demonstrate the approx. site of samples acquisition. Histologic and immunohistochemical scans show a representative cutout from the same position of the specimen for HE, EVG and various antibody stainings. For all histologic images the aortic lumen is oriented upwards. All samples are presented in the same orientation (A = anterior, P = posterior, R = right, L= left, H = head, T = tail) (scale bar 200µm). The antibody list can be found in **Suppl. Table I**.

**
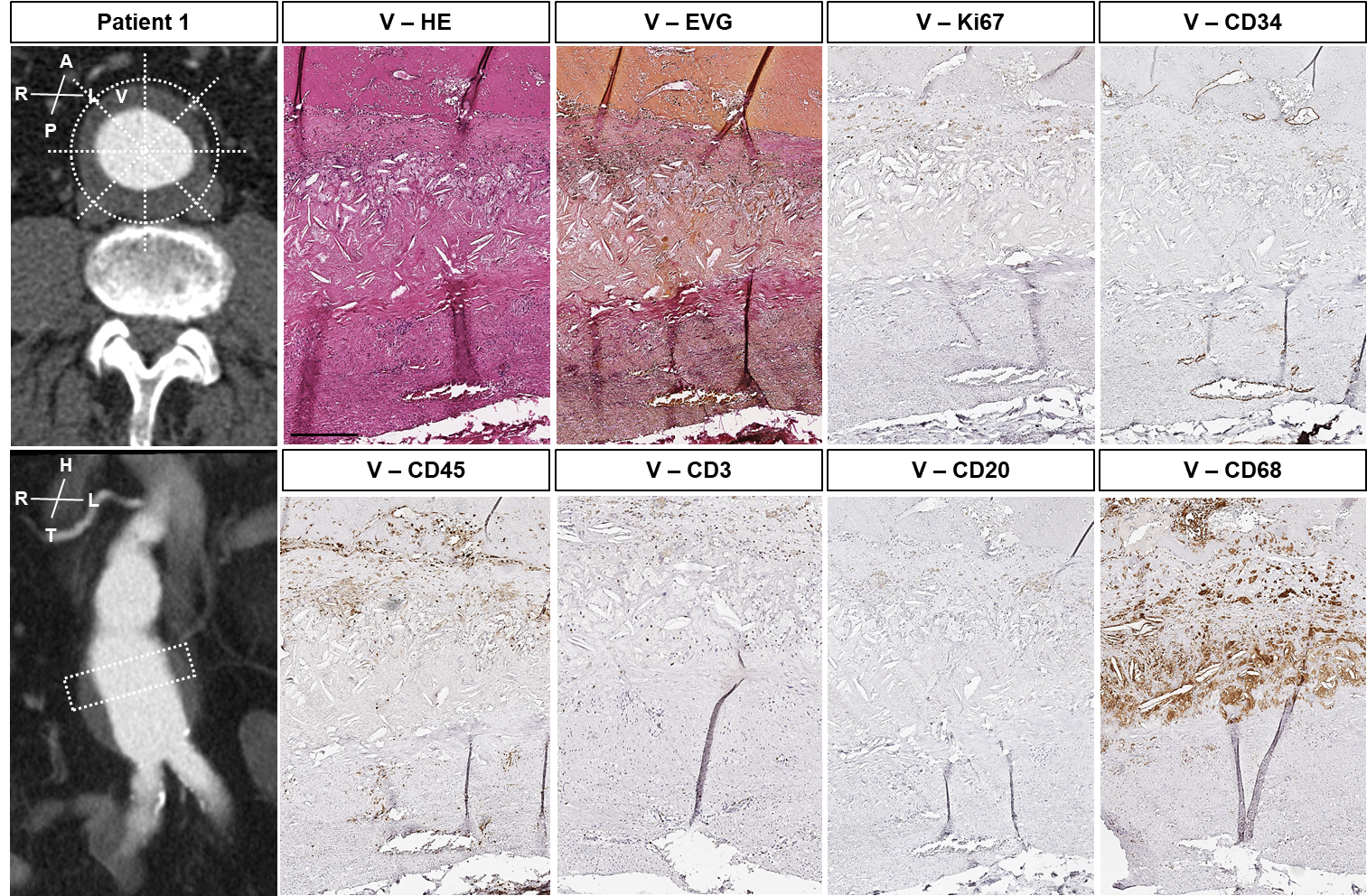
**

**Suppl. Figure 7: Patient 1 sample acquisition, histomorphology and immunohistochemistry V.** CT-angiogram and dotted lines demonstrate the approx. site of samples acquisition. Histologic and immunohistochemical scans show a representative cutout from the same position of the specimen for HE, EVG and various antibody stainings. For all histologic images the aortic lumen is oriented upwards. All samples are presented in the same orientation (A = anterior, P = posterior, R = right, L= left, H = head, T = tail) (scale bar 200µm). The antibody list can be found in **Suppl. Table I**.

**
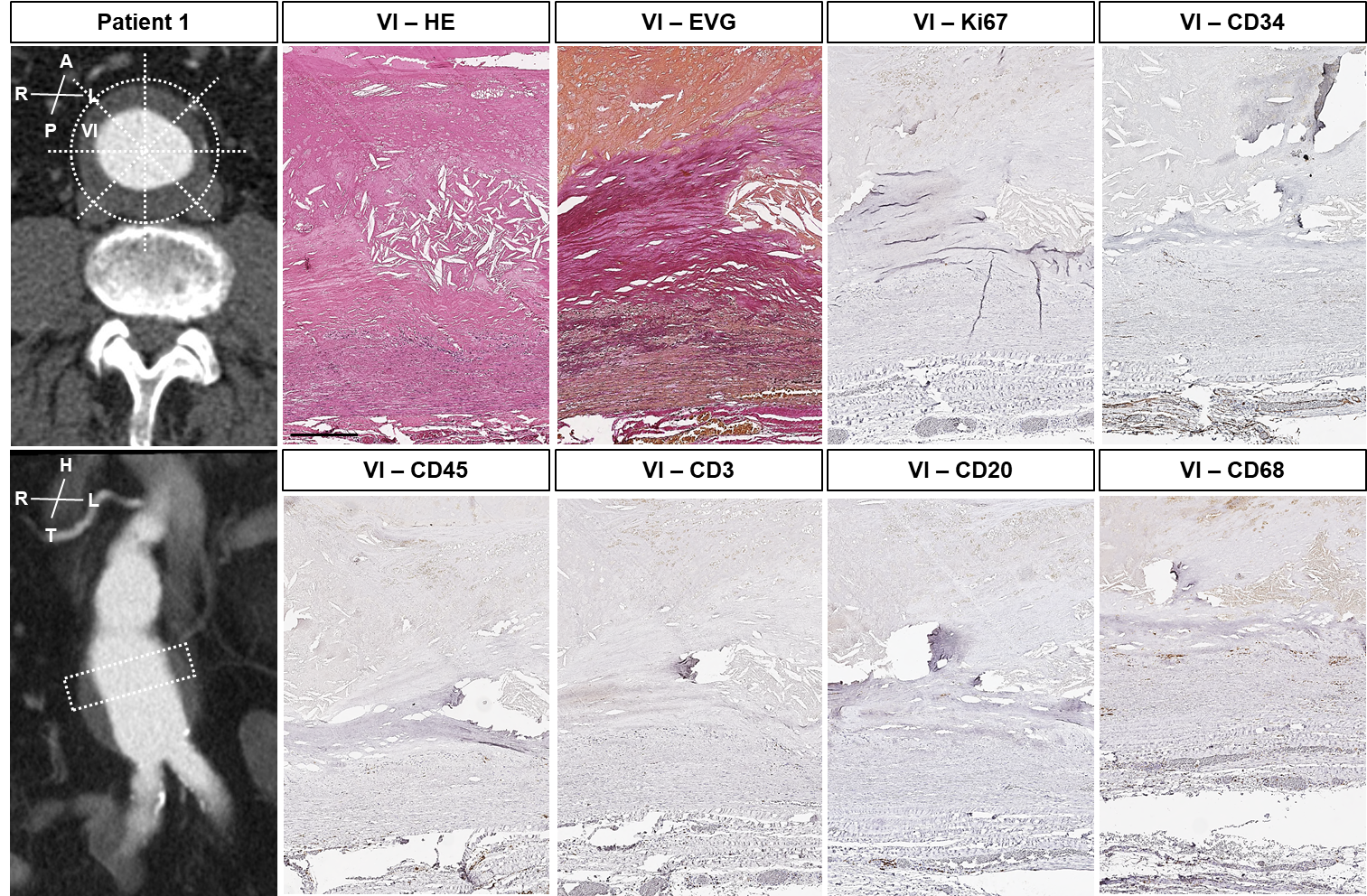
**

**Suppl. Figure 8: Patient 1 sample acquisition, histomorphology and immunohistochemistry VI.** CT-angiogram and dotted lines demonstrate the approx. site of samples acquisition. Histologic and immunohistochemical scans show a representative cutout from the same position of the specimen for HE, EVG and various antibody stainings. For all histologic images the aortic lumen is oriented upwards. All samples are presented in the same orientation (A = anterior, P = posterior, R = right, L= left, H = head, T = tail) (scale bar 200µm). The antibody list can be found in **Suppl. Table I**.

**
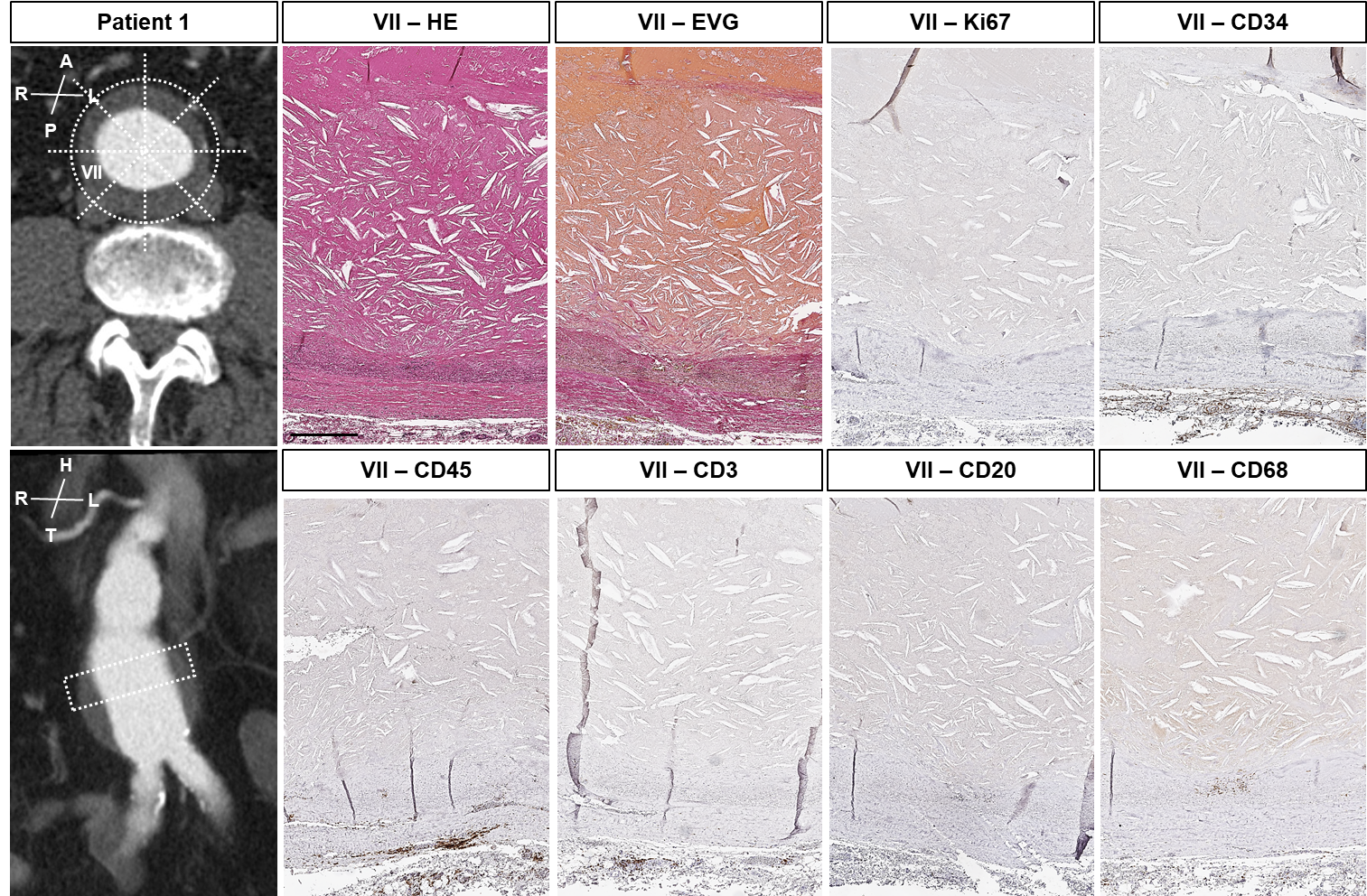
**

**Suppl. Figure 9: Patient 1 sample acquisition, histomorphology and immunohistochemistry VII.** CT-angiogram and dotted lines demonstrate the approx. site of samples acquisition. Histologic and immunohistochemistry photos show a representative cutout from the same position of the specimen for HE, EVG and various antibody stains. For all histologic photos the aortic lumen is oriented upwards. All samples are presented in the same orientation (A = anterior, P = posterior, R = right, L= left, H = head, T = tail) (scale bar 200µm). The antibody list can be found in **Suppl. Table I**.

**
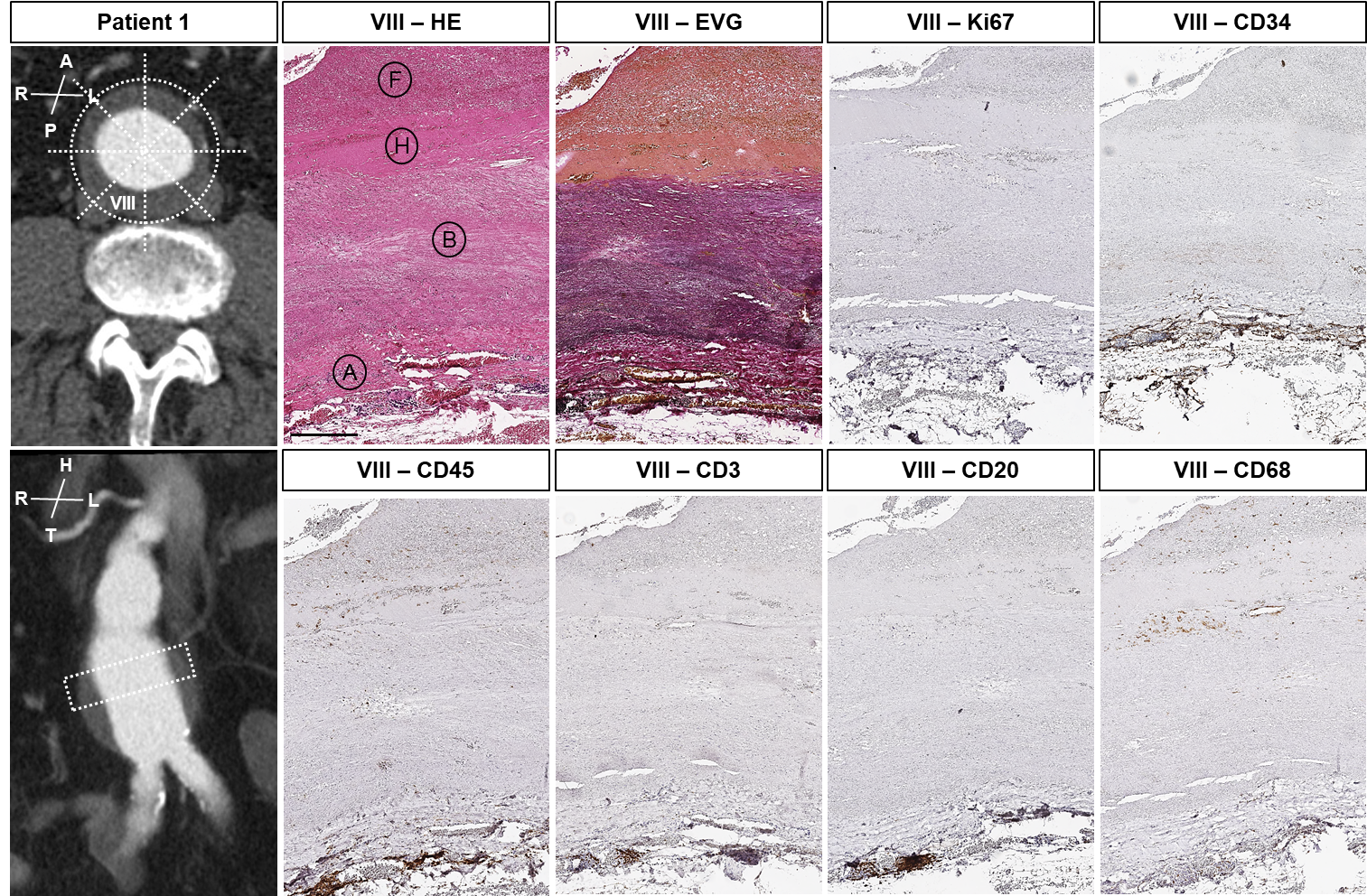
**

**Suppl. Figure 10: Patient 1 sample acquisition, histomorphology and immunohistochemistry VIII.** CT-angiogram and dotted lines demonstrate the approx. site of samples acquisition. Histologic and immunohistochemistry photos show a representative cutout from the same position of the specimen for HE, EVG and various antibody stains. For all histologic photos the aortic lumen is oriented upwards. All samples are presented in the same orientation (A = anterior, P = posterior, R = right, L= left, H = head, T = tail) (scale bar 200µm). The antibody list can be found in **Suppl. Table I**.

**
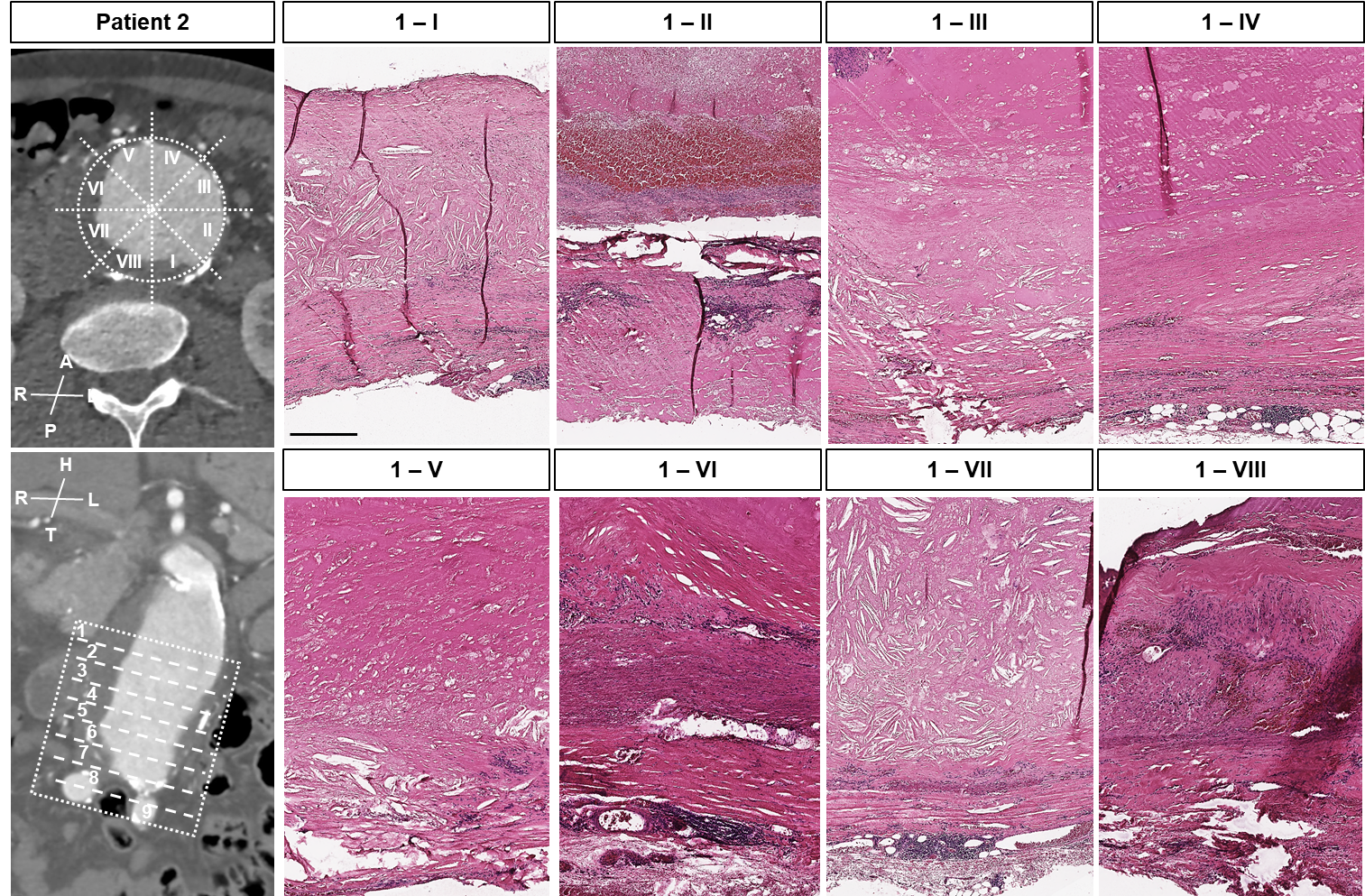
**

**Suppl. Figure 11: Patient 2 sample acquisition and histomorphology 1.** The dotted box and circle depict the specific termination for samples (1-9; I-VIII). Histologic images show eight (I-VIII) representative whole wall cutouts at level 1 (HE staining). Specific histologic features observed include: **(A)** elastic fibres, **(B)** collagen deposition, **(C)** calcification, **(D)** intramural bleeding, **(E)** inflammatory cell infiltration and **(F)** thrombus coverage and are depicted in detail in **Fig. 2**. All samples are presented in the same orientation (A = anterior, P = posterior, R = right, L= left, H = head, T = tail) (scale bar 250µm). For all histologic images, the aortic lumen is oriented upwards.

**
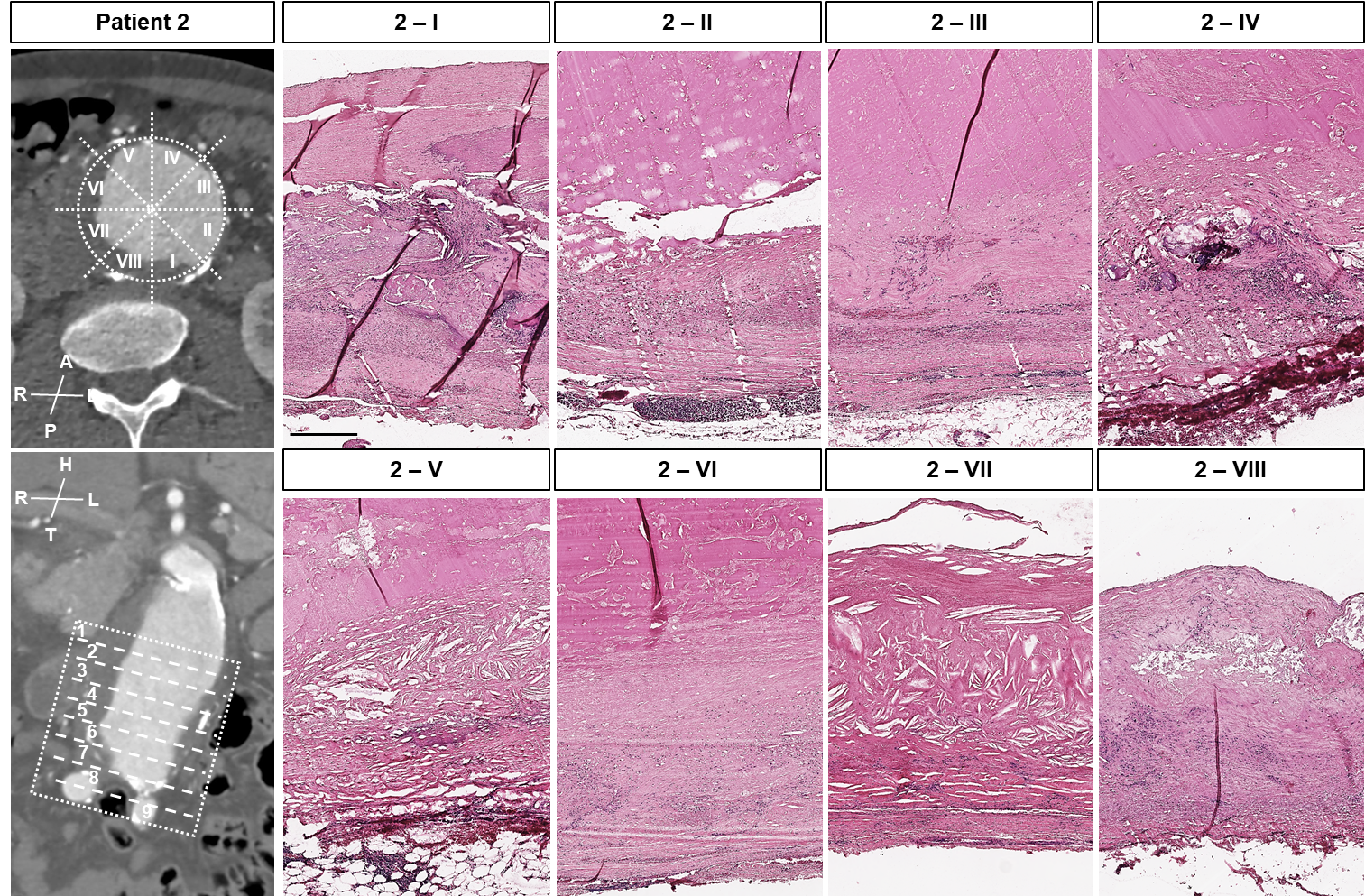
**

**Suppl. Figure 12: Patient 2 sample acquisition and histomorphology 2.** The dotted box and circle depict the specific termination for samples (1-9; I-VIII). Histologic photos show eight (I-VIII) representative whole wall cutouts at level 2 (HE staining). Specific histologic features observed include: **(A)** elastic fibres, **(B)** collagen deposition, **(C)** calcification, **(D)** intramural bleeding, **(E)** inflammatory cell infiltration and **(F)** thrombus coverage and are depicted in detail in **Fig. 2**. All samples are presented in the same orientation (A = anterior, P = posterior, R = right, L= left, H = head, T = tail) (scale bar 250µm). For all histologic images, the aortic lumen is oriented upwards.

**
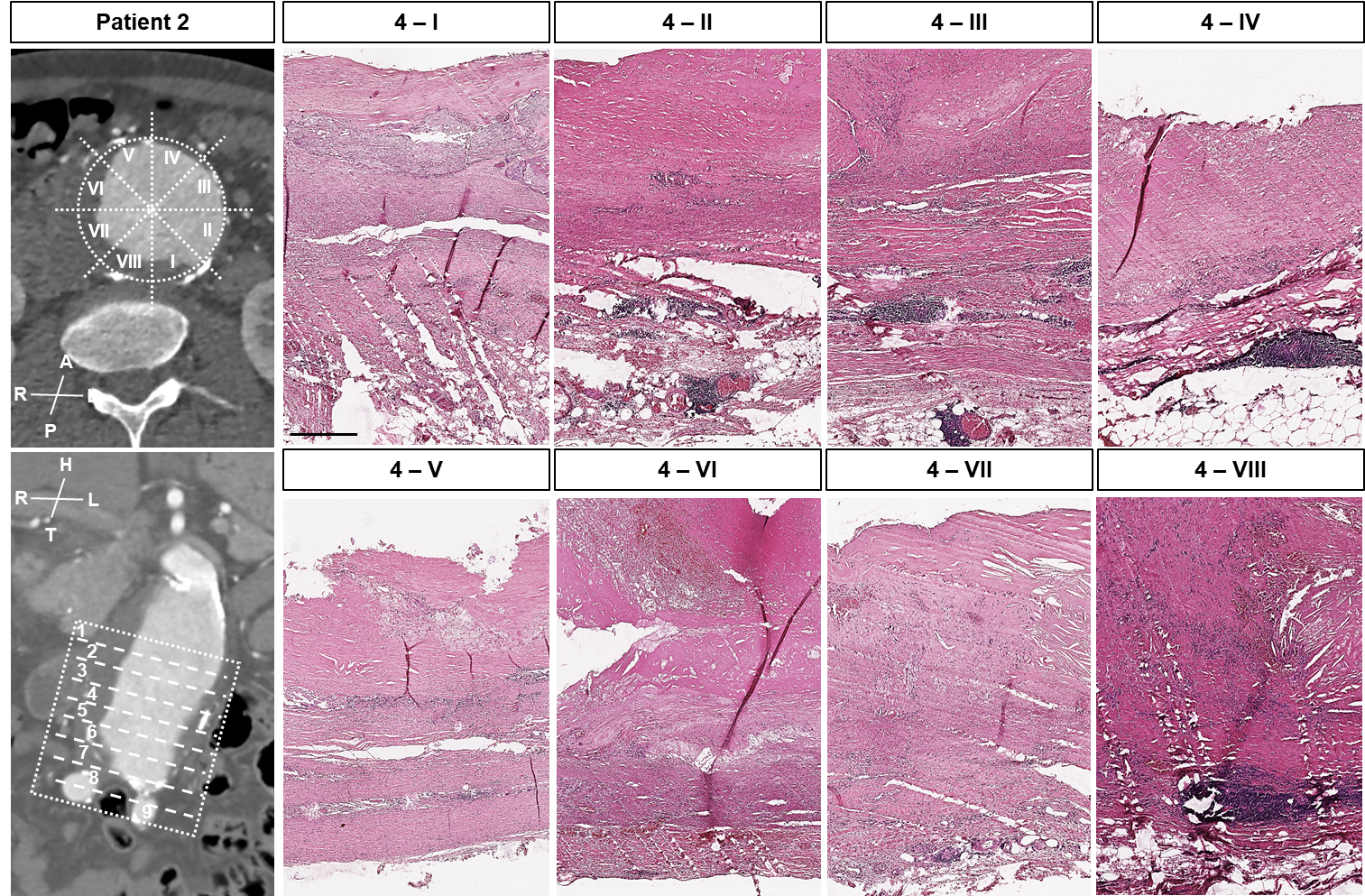
**

**Suppl. Figure 13: Patient 2 sample acquisition and histomorphology 4.** The dotted box and circle depict the specific termination for samples (1-9; I-VIII). Histologic photos show eight (I-VIII) representative whole wall cutouts at level 4 (HE staining). Specific histologic features observed include: **(A)** elastic fibres, **(B)** collagen deposition, **(C)** calcification, **(D)** intramural bleeding, **(E)** inflammatory cell infiltration and **(F)** thrombus coverage and are depicted in detail in **Fig. 2**. All samples are presented in the same orientation (A = anterior, P = posterior, R = right, L= left, H = head, T = tail) (scale bar 250µm). For all histologic imaegs, the aortic lumen is oriented upwards.

**
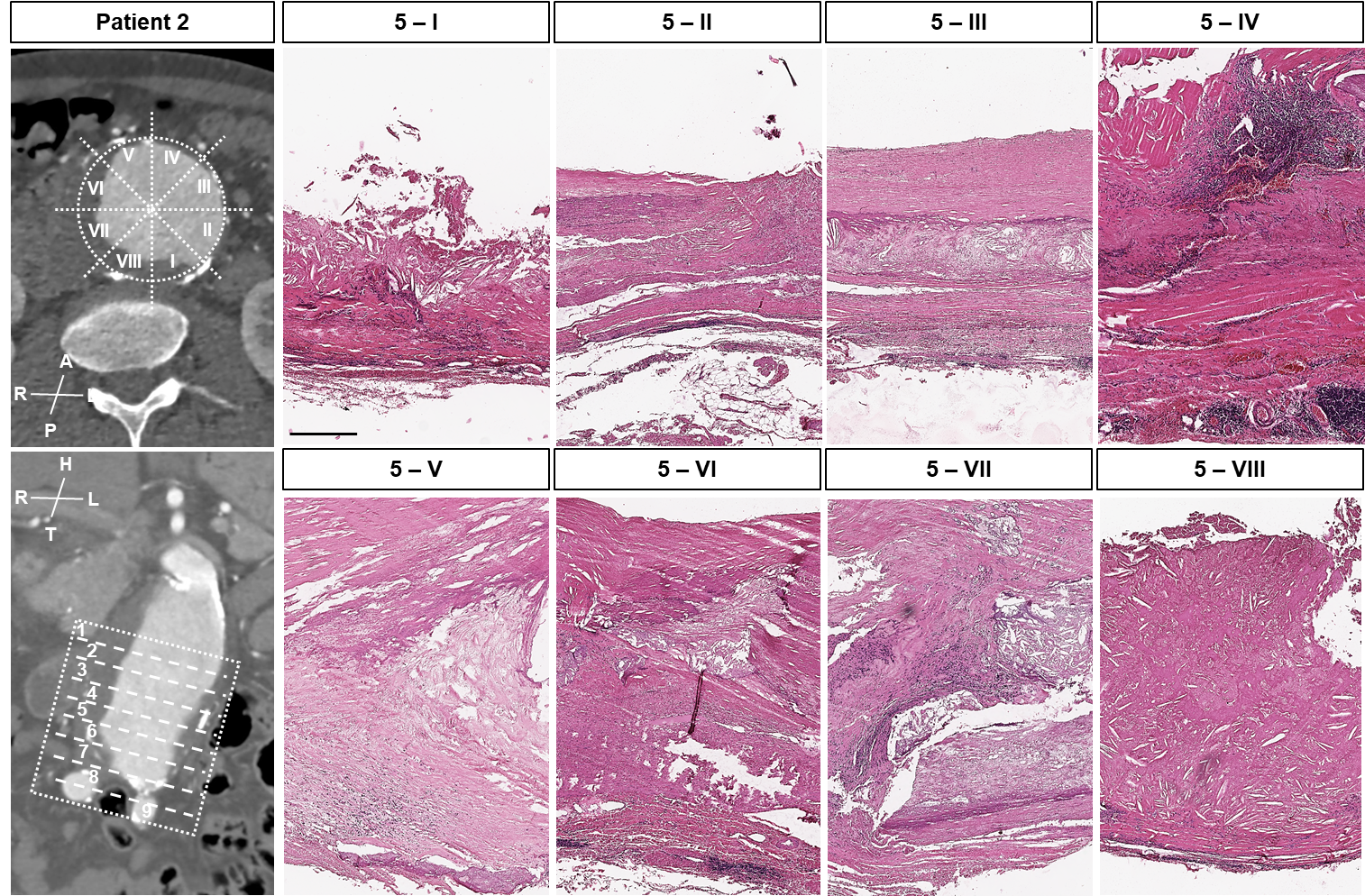
**

**Suppl. Figure 14: Patient 2 sample acquisition and histomorphology 5.** The dotted box and circle depict the specific termination for samples (1-9; I-VIII). Histologic photos show eight (I-VIII) representative whole wall cutouts at level 5 (HE staining). Specific histologic features observed include: **(A)** elastic fibres, **(B)** collagen deposition, **(C)** calcification, **(D)** intramural bleeding, **(E)** inflammatory cell infiltration and **(F)** thrombus coverage and are depicted in detail in **Fig. 2**. All samples are presented in the same orientation (A = anterior, P = posterior, R = right, L= left, H = head, T = tail) (scale bar 250µm). For all histologic images, the aortic lumen is oriented upwards.

**
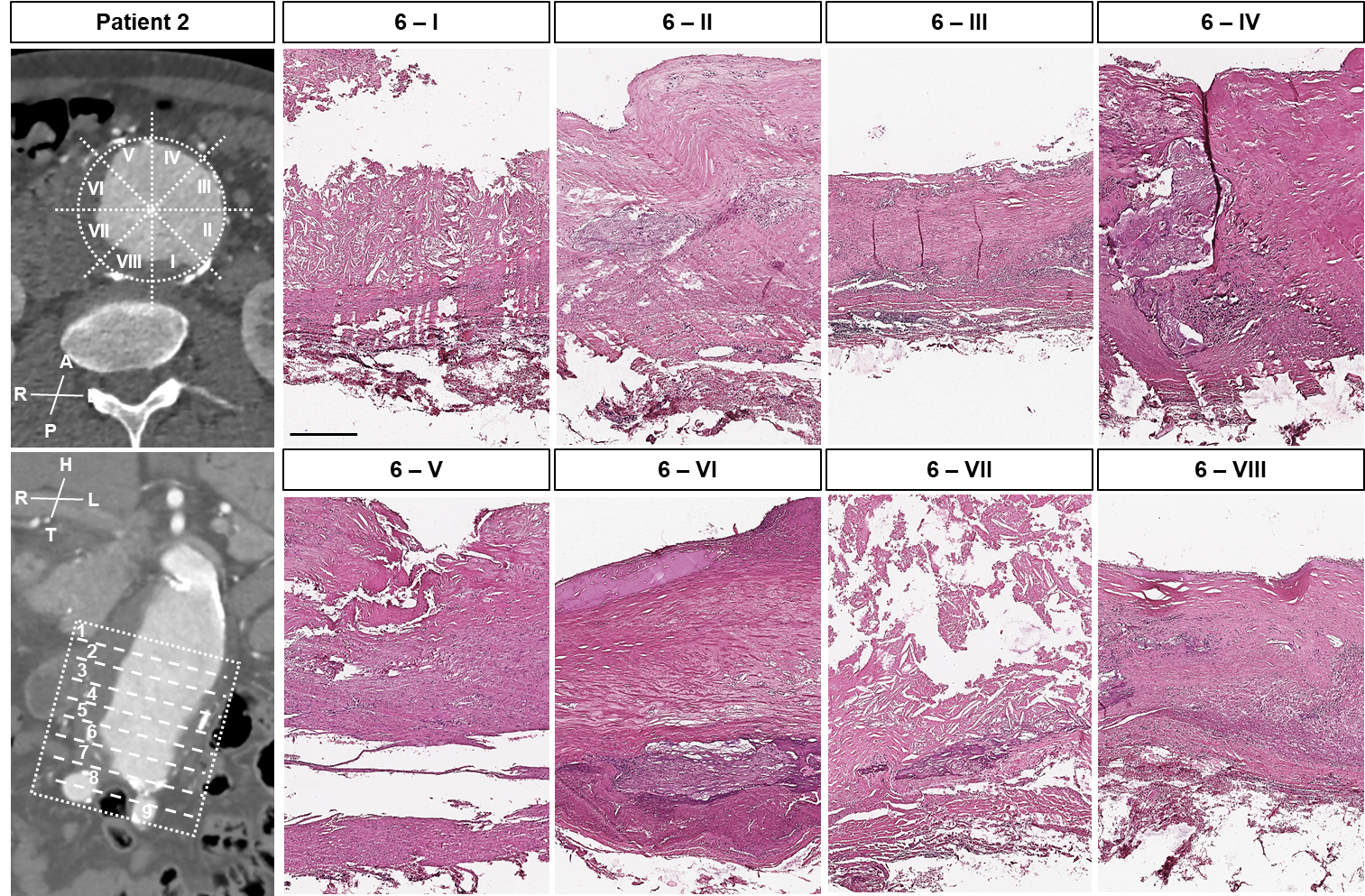
**

**Suppl. Figure 15: Patient 2 sample acquisition and histomorphology 6.** The dotted box and circle depict the specific termination for samples (1-9; I-VIII). Histologic photos show eight (I-VIII) representative whole wall cutouts at level 6 (HE staining). Specific histologic features observed include: **(A)** elastic fibres, **(B)** collagen deposition, **(C)** calcification, **(D)** intramural bleeding, **(E)** inflammatory cell infiltration and **(F)** thrombus coverage and are depicted in detail in **Fig. 2**. All samples are presented in the same orientation (A = anterior, P = posterior, R = right, L= left, H = head, T = tail) (scale bar 250µm). For all histologic photos, the aortic images is oriented upwards.

**
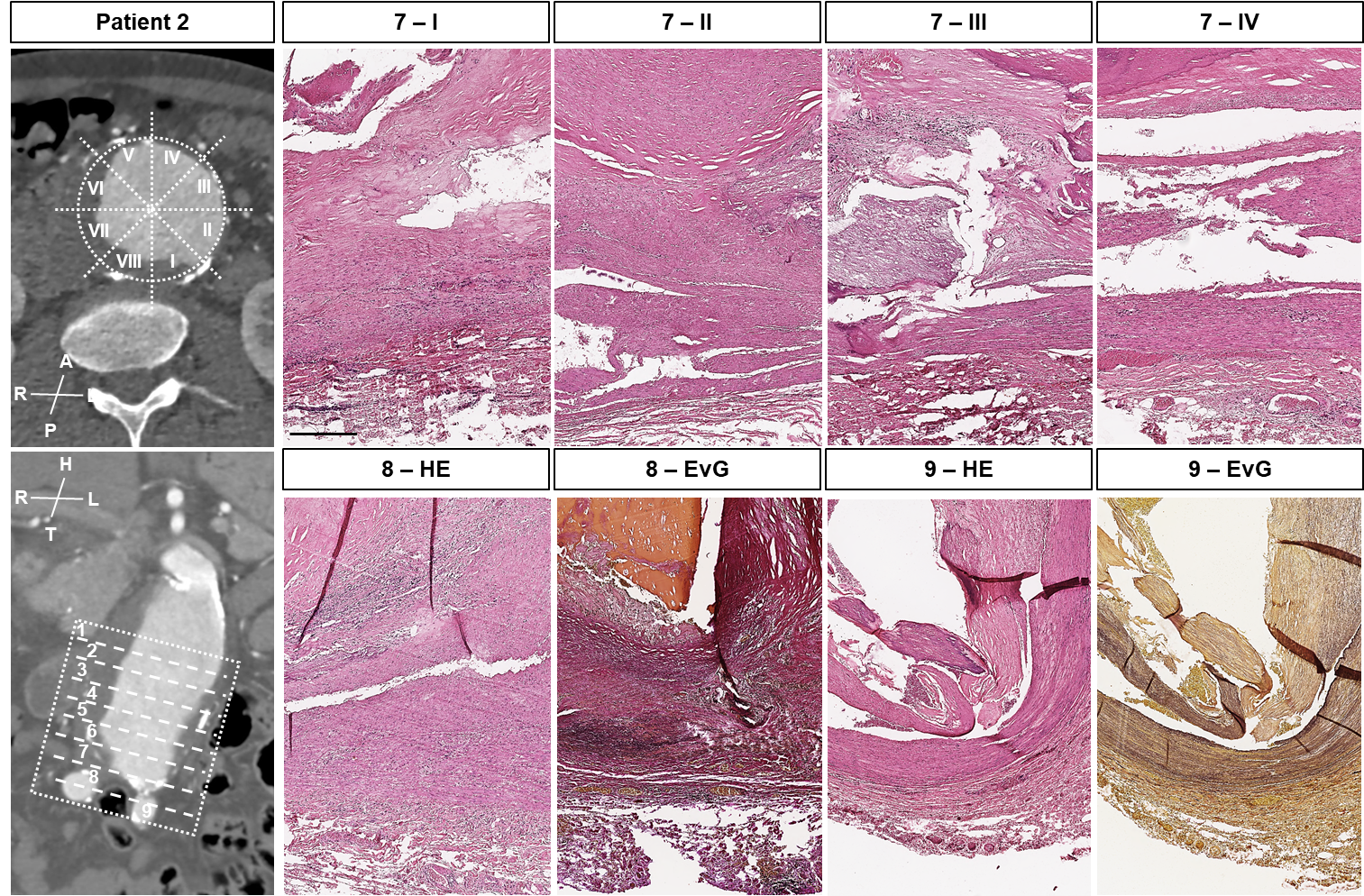
**

**Suppl. Figure 16: Patient 2 sample acquisition and histomorphology 7.** The dotted box and circle depict the specific termination for samples (1-9; I-VIII). Histologic photos show eight (I-VIII) representative whole wall cutouts at levels 7-9 (HE staining). Specific histologic features observed include: **(A)** elastic fibres, **(B)** collagen deposition, **(C)** calcification, **(D)** intramural bleeding, **(E)** inflammatory cell infiltration and **(F)** thrombus coverage and are depicted in detail in **Fig. 2**. All samples are presented in the same orientation (A = anterior, P = posterior, R = right, L= left, H = head, T = tail) (scale bar 250µm). For all histologic images, the aortic lumen is oriented upwards.

**
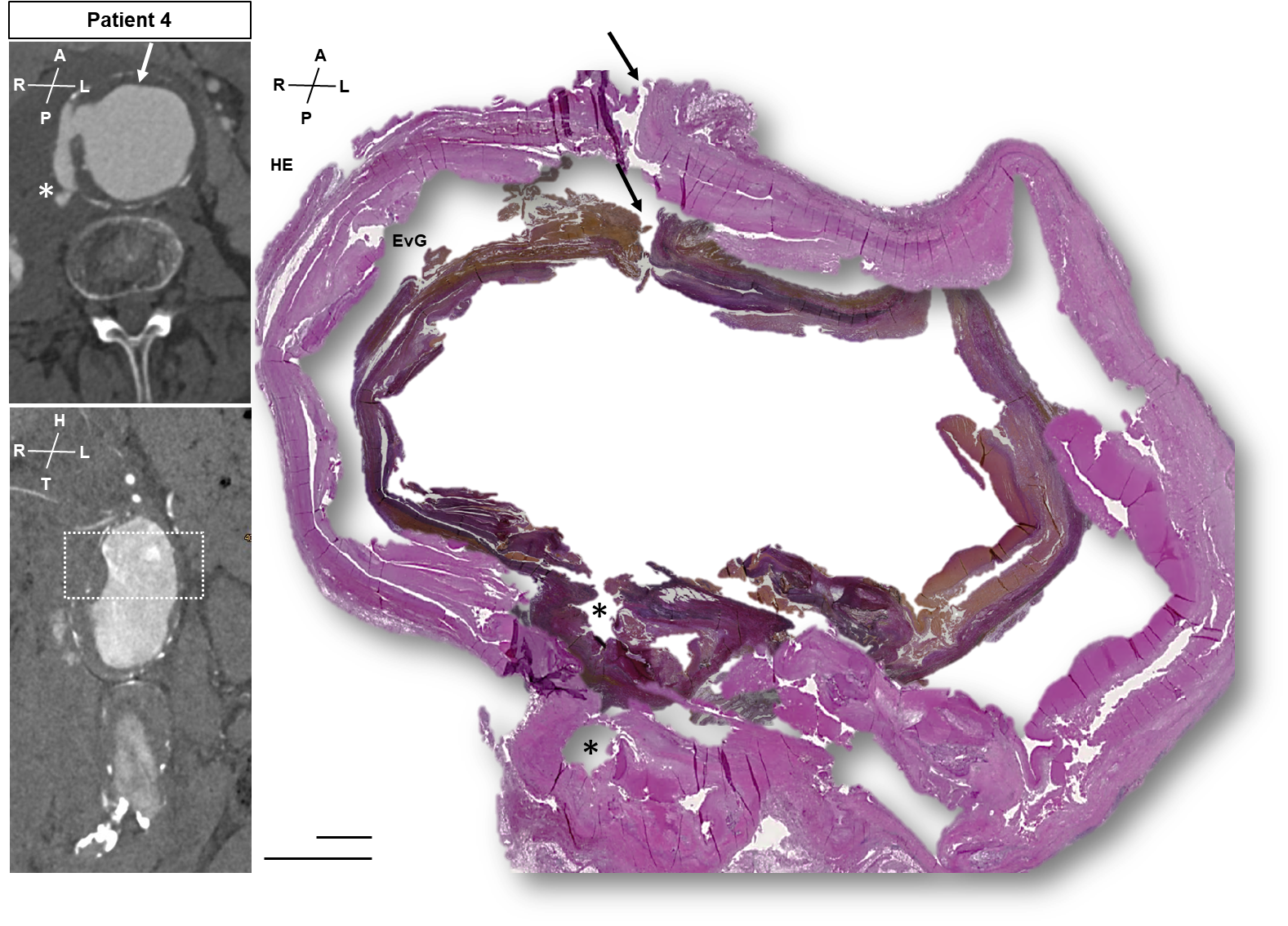
**

**Suppl. Figure 17: Patient 4 sample acquisition and histomorphology.** CT-angiogram and dotted lines demonstrate the approx. site of samples acquisition. The arrow marks the aneurysm sac incision during open repair. The histologic images show a complete circular preparation of the aneurysm sac for HE (outer ring) and EVG staining (inner ring). The asterisk marks the potential position of the rupture site. Samples are oriented in line with the CT-angiogram (A = anterior, P = posterior, R = right, L= left, H = head, T = tail) (scale bar 5mm each, upper bar = inner photo). (scale bar 500µm)

**
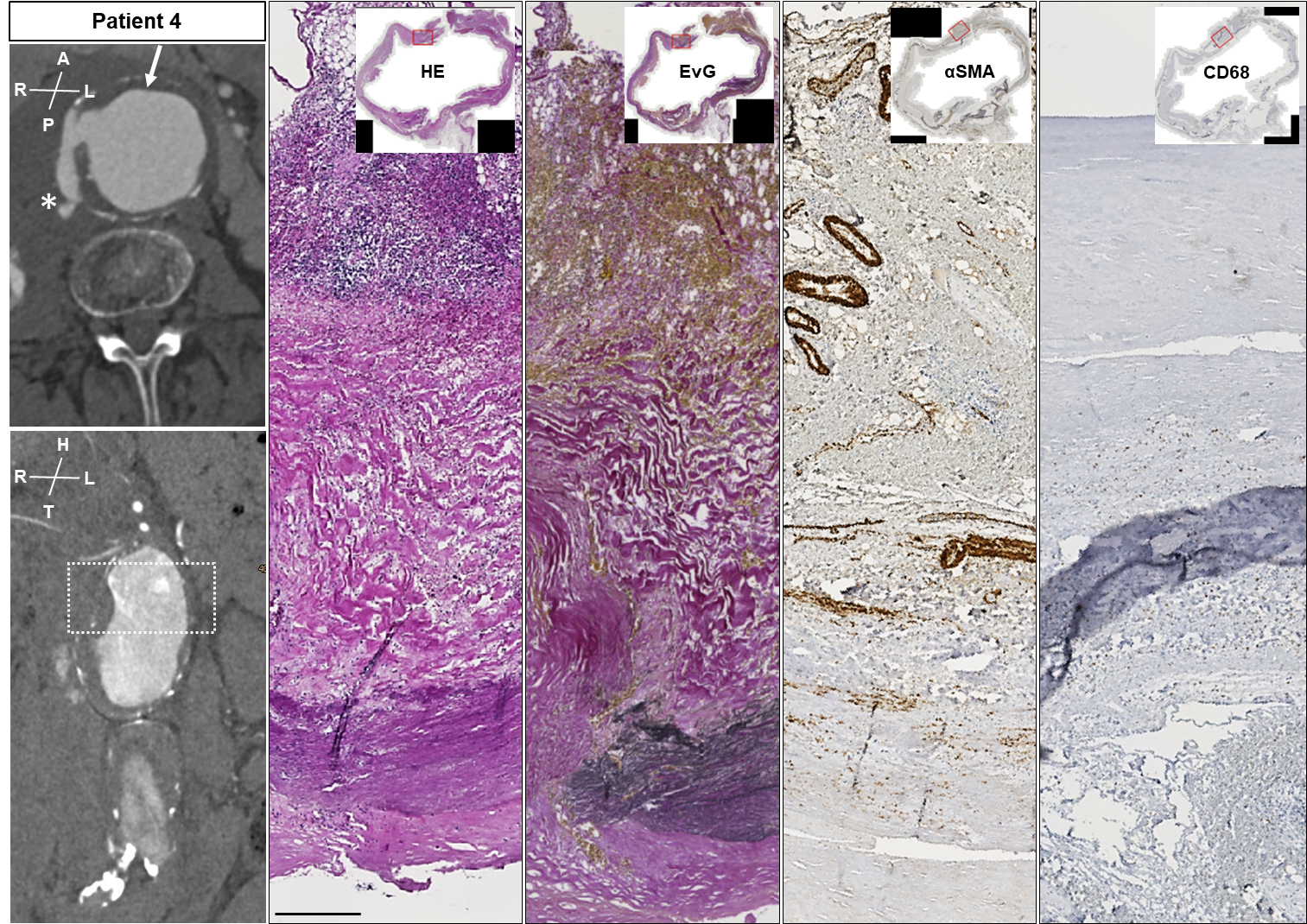
**

**Suppl. Figure 18: Patient 4 sample acquisition, histomorphology and immunohistochemistry 1.** CT-angiogram and dotted lines demonstrate the approx. site of samples acquisition. The arrow marks the aneurysm sac incision during open repair. The asterisk marks the potential direction of the rupture site. The histologic scans show a cutout from complete circular preparation of the aneurysm sac for HE and EVG staining. Additionally, immunohistochemistry for alpha smooth muscle actin (αSMA) and CD68 has been performed and is shown as cutout and whole slide inlay image. For all histologic images the aortic lumen is oriented upwards. All samples are presented in the same orientation (A = anterior, P = posterior, R = right, L= left, H = head, T = tail) (scale bar 400µm).

**
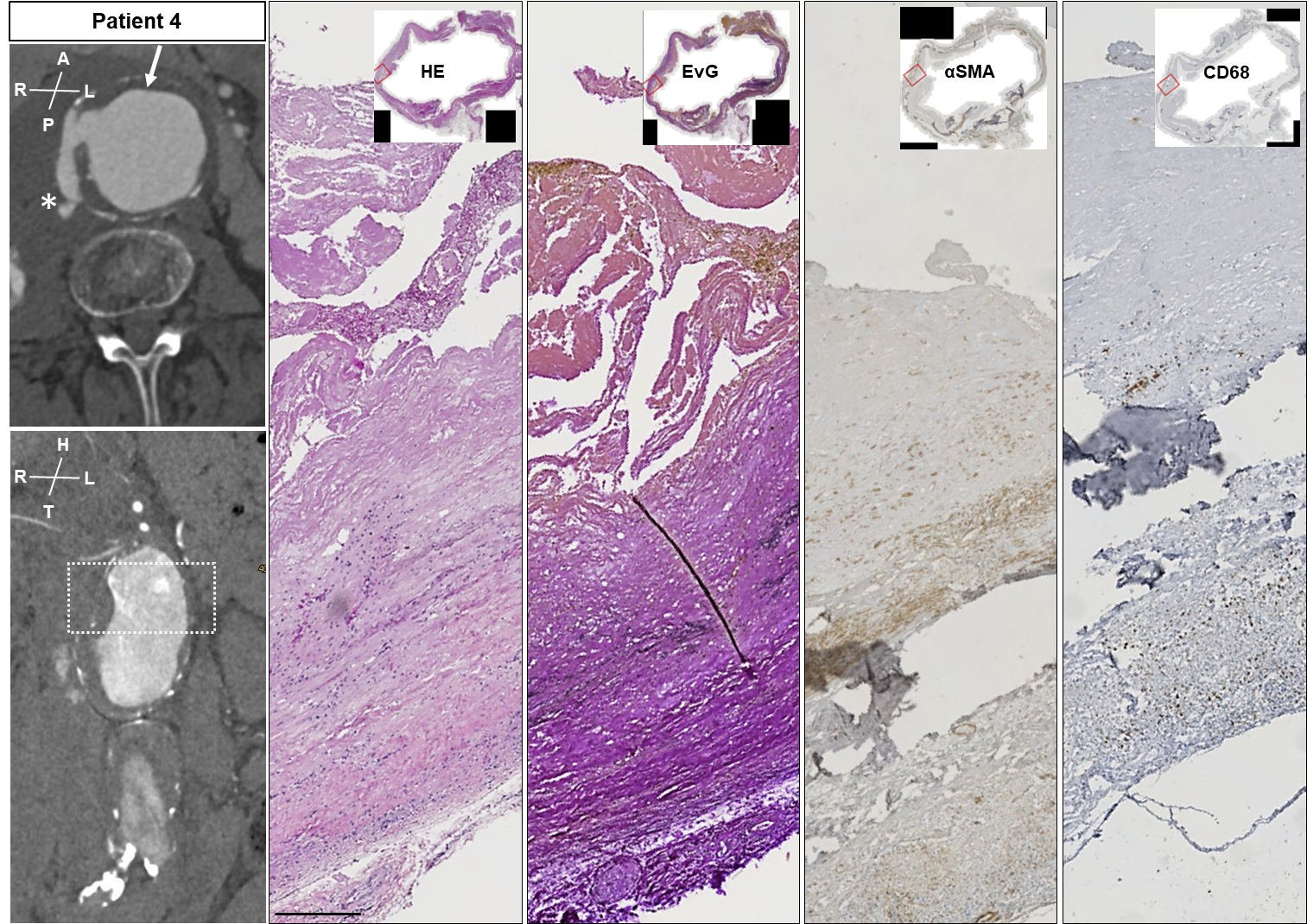
**

**Suppl. Figure 19: Patient 4 sample acquisition, histomorphology and immunohistochemistry 2.** CT-angiogram and dotted lines demonstrate the approx. site of samples acquisition. The arrow marks the aneurysm sac incision during open repair. The asterisk marks the potential direction of the rupture site. The histologic scans show a cutout from complete circular preparation of the aneurysm sac for HE and EVG staining. Additionally, immunohistochemistry for alpha smooth muscle actin (αSMA) and CD68 has been performed and is shown as cutout and whole slide inlay image. For all histologic images the aortic lumen is oriented upwards. All samples are presented in the same orientation (A = anterior, P = posterior, R = right, L= left, H = head, T = tail) (scale bar 400µm).

**
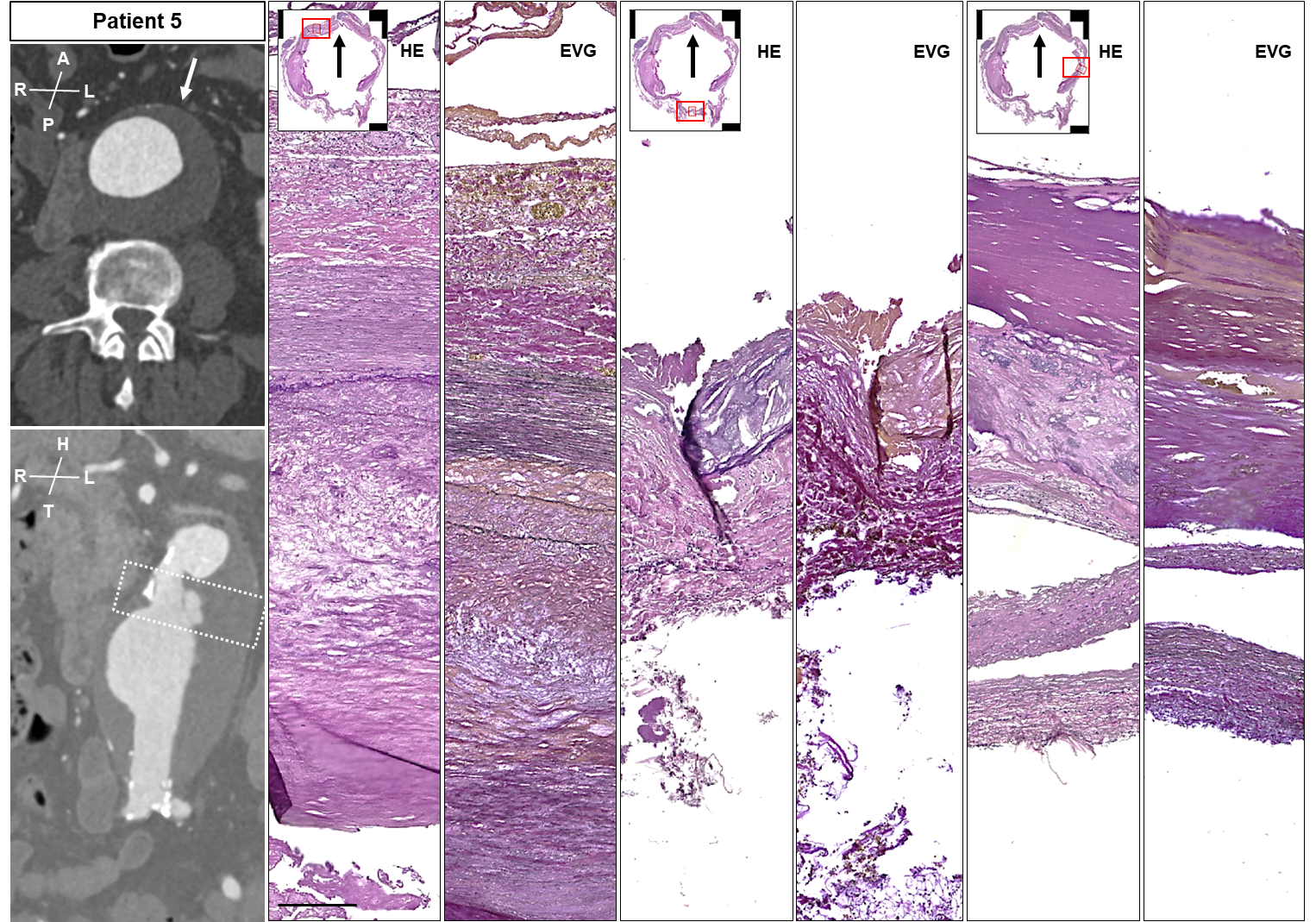
**

**Suppl. Figure 20: Patient 5 sample acquisition and histomorphology.** CT-angiogram and dotted lines demonstrate the approx. site of samples acquisition. The arrow marks the aneurysm sac incision during open repair. The histologic photos show a cutout from complete circular preparation of the aneurysm sac for HE and EvG staining at three different localizations. For all histologic images the aortic lumen is oriented upwards. (A = anterior, P = posterior, R = right, L= left, H = head, T = tail) ) (scale bar 100µm).
